# Online Information-Seeking Behaviour for Major Cancers: A 10-Year Infodemiological Study to Inform Public Health and Health Promotion Priorities in Hungary

**DOI:** 10.3390/medsci14060681

**Published:** 2026-10-17

**Authors:** Márió Gajdács, Irena Ilić, Dóra Szabó-Kurucz, Regina Molnár, Edit Paulik, Diána Elmer, Tímea Csákvári, Zsuzsanna Kívés

**Affiliations:** 1 Department of Public Health, Albert Szent-Györgyi Medical School, University of Szeged, 6720 Szeged, Hungary; szabo-kurucz.dora@szte.hu (D.S.-K.); molnar.regina@med.u-szeged.hu (R.M.); paulik.edit@med.u-szeged.hu (E.P.); 2 MTA-SZTE Lendület “Momentum” Anthropogenic Stress and Plant Resilience Research Group, 6726 Szeged, Hungary; 3 Faculty of Medicine, University of Belgrade, 11000 Belgrade, Serbia; irena.ilic@med.bg.ac.rs; 4 Institute of Health Insurance, Faculty of Health Sciences, University of Pécs, 7621 Pécs, Hungary; diana.elmer@etk.pte.hu (D.E.);; 5 Department of Health Economics and Health Care Management, Institute of Health Insurance, Faculty of Health Sciences, University of Pécs, 8900 Zalaegerszeg, Hungary; timea.csakvari@etk.pte.hu

**Keywords:** infodemiology, Google Trends, cancer, public health, educational campaigns, information-seeking, health communication, COVID-19

## Abstract

**Background/Objectives:** To analyse trends in online information-seeking behaviour for five major cancers in Hungary, compare them with global trends, and assess changes during the COVID-19 pandemic, cancer-related awareness campaigns, and major cancer-related events on search activity. **Methods:** A retrospective observational infodemiological study was conducted using Google Trends™ (GT) data for lung, colorectal, breast, prostate, and pancreatic cancers between 2013 and 2022. Search intensity was assessed using uncorrected relative search volume (RSV_raw_) as the primary measure, complemented by RSV_sens_, as an Internet penetration-adjusted sensitivity analysis. Descriptive statistics, parametric tests, time-series and seasonality analyses, Mann–Kendall tests, and joinpoint regression were performed using IBM SPSS Statistics 22.0, PAST 4.03, and Joinpoint 5.3.0 (α: 0.05; 95% confidence interval [CI]). Benjamini–Hochberg (BH) false discovery rate (FDR) correction was applied for multiple testing. **Results:** Among the cancer types relevant to our study, lung and breast cancer showed the highest reference-adjusted RSV (RSV*_ref_*: 61) in Hungary, followed by prostate (RSV*_ref_*: 59), colorectal (RSV*_ref_*: 52), and pancreatic cancer (RSV*_ref_*: 11); whereas globally, breast cancer ranked first (RSV*_ref_*: 49). RSV_sens_ showed significant decreasing trends for all five cancer types (Mann–Kendall, all *p* < 0.05), with average annual percentage changes (AAPCs) of −5.39% for lung, −4.06% for breast, −3.56% for colorectal, −3.59% for prostate, and −5.23% for pancreatic cancer, respectively (all *p* < 0.05). Breast and prostate cancers exhibited significant (*p* < 0.001) and recurring seasonal peaks. During COVID-19 (vs. the pre-pandemic period), mean RSV values decreased by 10–30% across all cancers; only colorectal cancer-related campaigns were associated with a notable increase in search intensity (+66.9%, *p* = 0.013, *q* = 0.058), although this association did not remain significant after FDR correction.. Symptom-related searches predominated, whereas screening- and prevention-related queries were infrequent during the study period. **Conclusions:** GT may be a useful complementary tool for monitoring public interest and evaluating public health communication. The absence of consistent increases in search activity during most campaign periods, together with the predominance of symptom-related queries, suggests that current communication initiatives may have limited measurable visibility in GT search behaviour and highlights the need to strengthen communication on cancer prevention and early detection.

## 1. Introduction

Malignant disorders are a major public health burden globally and cancer was the second leading cause of death worldwide in 2023, accounting for an estimated 10.4 million deaths and 271 million disability-adjusted life-years (DALYs) [1]. While cancers affect people in all socio-economic strata, low- and middle-income regions bear a disproportionately high burden of both new cancer cases and cancer-related deaths, a tendency which is expected to be maintained in the coming decades [1,2]. According to the World Health Organization (WHO) Global Cancer Observatory, in 2022, an estimated 19,965,054 incident cancer cases were reported; among women, breast (23.8%), lung (9.4%), and colorectal cancer (8.9%) were the most frequently diagnosed malignancies, whereas lung (15.3%), prostate (14.2%), and colorectal cancer (10.4%) predominated among men. In the same year, cancer accounted for 9,736,520 deaths. Among women, breast (15.4%), lung (13.6%), and colorectal cancer (9.4%) were the leading causes of cancer-related mortality, while among men, lung (22.7%), liver (9.6%), and colorectal cancer (9.2%) caused the highest number of cancer-related deaths [3].

According to preliminary data from the Hungarian National Cancer Registry, operated by the National Institute of Oncology [4], 70,816 incident cases of cancer (excluding non-melanoma skin cancer) were recorded in Hungary in 2022, remaining below the pre-pandemic level of 74,249 cases recorded in 2019 [4,5]. According to age-standardized estimates of the European Cancer Information System (ECIS), the overall cancer incidence rate in Hungary was 626/100,000 population in 2022 [5]. Within the Hungarian context, prostate cancer (165 cases/100,000 population; 21.0%), colorectal cancer (138/100,000; 18.0%), and lung cancer (139/100,000; 18.0%) were the most common malignancies among males, whereas breast cancer (136/100,000; 26.0%), colorectal cancer (68/100,000; 13.0%), and lung cancer (72/100,000; 14.0%) predominated among women. In the same year—with the exception of breast cancer—the incidence of these major cancer types exceeded the European Union (EU_27_) average. For example, among men, the incidence rates of lung and colorectal cancer were 139 and 138/100,000 in Hungary, compared with 95 and 93/100,000 in the EU_27_, respectively; among women, the corresponding rates were 72 vs. 44/100,000 for lung cancer and 68 vs. 58/100,000 for colorectal cancer [5].

The five cancer types included in the present analysis were selected a priori, based on their public health relevance in Hungary, rather than on the extent of online information-seeking intensity associated with them in this country. Lung, colorectal, breast and prostate cancers were selected, because they represent some of the most important malignancies in terms of incidence and/or mortality in Hungary, while breast and colorectal cancers are also notable, due to directly being linked to organized population-based screening programmes [5,6]. On the other hand, pancreatic cancer was included, due to its poor prognosis, limited opportunities for early detection and occurrence of short-term media-driven peaks in pancreatic-cancer related public attention [7]. Thus, the selected cancers allowed us to examine online information-seeking across malignancies with different epidemiological burdens, screening relevance, preventability and prognosis.

In the context of mortality, lung cancer (78 deaths/100,000 population), colorectal cancer (50/100,000), and breast cancer (23/100,000) were the leading causes of cancer-related mortality in Hungary in 2021. Despite the observable declining trend in cancer-related deaths in Hungary, overall cancer mortality exceeded the EU_27_ average by 32.0% [5]. However, it must be noted that the persistently and exceedingly high mortality from lung cancer reported—compared with other European countries—may be partly attributable to Hungary’s exceptionally high autopsy rate. In 2019, the autopsy rate for all deaths was 35.2% in Hungary, compared with an EU_27_ average of 12.1%, which may increase the probability of identifying previously undiagnosed lung cancer cases at autopsies [6].

Cancers represent a highly heterogeneous group of diseases, with substantial differences in their biological characteristics, treatment response, and clinical outcomes [8]. This heterogeneity is also reflected in prognosis: the five-year relative survival exceeds 90.0% for several malignancies, when diagnosed at a localized (non-invasive) stage, including breast and prostate cancer, whereas survival remains substantially poorer for other malignancies, particularly when diagnosed at an advanced stage [9]. Pancreatic cancer represents one of the most striking examples of a malignancy with poor prognosis: approximately 85.0% of pancreatic ductal adenocarcinomas are diagnosed at an advanced stage, and are therefore ineligible for potentially curative surgical resection, with five-year survival remaining approximately 13.0% [10]. In Hungary, pancreatic cancer was the fifth leading cause of cancer-related death among men and the fourth among women in 2019 [11]. Although some non-modifiable characteristics (genetic determinants, age, sex), as well as occupational and environmental exposures play a substantial role in the development of cancers, behavioural and lifestyle-related risk factors (i.e., tobacco consumption, alcohol use, overweight and obesity, unhealthy diet and low physical activity) are key within the context of preventable cancer burden [12]. Furthermore, for several cancer types associated with substantial mortality, effective screening modalities are available, allowing for the detection of precancerous lesions or cancers at an early stage [13].

Participation in organized cancer screening programmes remains low in Hungary, particularly for breast and colorectal cancer screening [5,14,15]. Colorectal cancer is particularly relevant from a prevention and early-detection perspective, as the two-stage screening strategy utilized in most countries may contribute to the detection of both early-stage cancers and precancerous lesions [13]. In addition, a recent systematic review by Leonforte et al. has also highlighted that in selected, high-risk populations, such as kidney transplant recipients, evidence also supports risk-tailored, lifelong colonoscopy-based surveillance, particularly in younger individuals or those otherwise considered as high-risk recipients [16]. Therefore, colorectal cancer provides an important example for examining whether online information-seeking reflects public interest in screening, prevention and early detection.

Moreover, nationwide data from the Hungarian National Health Insurance Fund (NHIF) demonstrated a substantial decline in screening participation during the COVID-19 pandemic: mammography coverage decreased from 48.1 to 51.5% in 2012–2019 to 31.8% in the 2020–2021 screening cycle, while colorectal screening participation also decreased markedly in 2020 and 2021 [14,15]. During the initial phase of the pandemic, organized population-based breast and colorectal cancer screening programmes were temporarily suspended in Hungary, while elective and one-day surgical procedures were also restricted; oncological diagnostic and curative services, however, were formally exempt from these restrictions [17]. The disruption of screening was accompanied by a substantial reduction in mammography examinations and a subsequent decline in newly diagnosed breast cancer cases, indicating that pandemic-related changes in healthcare utilization may have delayed the detection of some cancers [17,18]. On the other hand, studies from different geographical contexts have demonstrated suboptimal levels of awareness among the general population regarding the main risk factors, warning signs, symptoms, and preventive strategies related to common cancers, even though Internet access were readily available [19,20,21,22]. This is particularly concerning given the considerable preventable component of the global cancer burden. According to recent global estimates, 37.8% of all new cancer cases worldwide in 2022, corresponding to approximately 7.1 million cases, were attributable to modifiable risk factors [23].

The Internet—and online sources more broadly—has become one of the primary sources of health information for the general population [24]. According to the WHO, the rapid expansion of the digital information environment has contributed to an “infodemic”, characterized by an overabundance of both accurate and misleading information (i.e., misinformation, malinformation and disinformation), making it increasingly difficult for individuals to identify trustworthy health-related information and make informed health decisions [25]. Consequently, monitoring online health information-seeking behaviour has become an important tool for public health surveillance, and has contributed to the emergence of the field of infodemiology [26,27]. Infodemiology has been defined as the study of the distribution and determinants of information in electronic media, particularly the Internet, with the aim of informing public health and public policy [26]. In this context, patterns of Internet searches, or the analysis of online search intensity, may provide population-level insights into information needs, concerns, and changes in health-related information-seeking behaviour over time [26,27].

Accordingly, infodemiological studies often distinguish between analysis of the supply side of online health information (“supply metrics”), referring to the nature and volume of information available on the Internet on a given topic, and the demand side (“demand metrics”), associated with the analysis of the public’s health information-seeking behaviour (e.g., online search intensity). Although information-seeking is a complex phenomenon, evaluating the intensity of online search behaviour may be used as a proxy measure to identify knowledge gaps, patient preferences, fears and unmet information needs at the population level, all of which may be relevant for health policy planning and to identify “best-buy” interventions in a regional or national context [26,27].

Although health information is available across numerous digital platforms, the use of online search engines through specific keywords and search terms is often a primary avenue through which users access websites and other online information sources. The vast majority of general web searches are still conducted through the Google^TM^ search engine, which maintained a global market share of approximately 90% or more throughout recent years [28]. Associated with this search engine, Google Trends^TM^ (GT) is an open platform, allowing for the assessment of population interest in specific topics over time. Consequently, GT may be used as a valuable tool for monitoring online search activity, although it should complement rather than replace traditional epidemiological data sources [26,29,30]. The global burden of cancer among adolescents and young adults is projected to increase by approximately 12.0% between 2022 and 2050. Although a 10.7% decline is expected in countries with a very high Human Development Index (HDI), the overall increase will be driven primarily by low-HDI countries, where both cancer incidence and mortality are projected to nearly double [31]. At the same time, online health information seeking has become especially widespread among young adults, further emphasizing the importance of investigating the digital health information environment as the future cancer burden continues to increase [32,33].

Previous infodemiological studies have demonstrated that Google^TM^ search activity related to cancer is sensitive to changes in disease incidence, awareness campaigns, media coverage, and public health emergencies, such as the COVID-19 pandemic, although these associations vary across cancer types, socio-economic and geographical settings [34,35,36,37,38,39]. Nevertheless, evidence remains limited regarding the extent to which public online interest reflects the actual cancer burden, responds sustainably to awareness campaigns, or is influenced by high-profile cancer-related events, particularly in Central and Eastern Europe (CEE). To date, little is known about how online information-seeking patterns related to major cancers have evolved in Hungary over the past decade, or which specific public information needs they reflect. This question is particularly relevant given recent Hungarian evidence demonstrating substantial geographical inequalities in cancer incidence and mortality, suggesting unmet needs in cancer prevention and healthcare delivery [40]. However, it remains unknown to what extent behaviour reflects these public health priorities.

The present study adopts the latter perspective/strategy, by evaluating the extent to which online searches related to cancer reflect public information needs and their temporal changes. Accordingly, the aim of the present study was to analyse the patterns and nature of online information-seeking related to cancers of notable public health importance (i.e., lung, colorectal, breast, prostate, and pancreatic cancers) in Hungary between 2013 and 2022, and to compare and contrast these findings with global trends. Furthermore, we evaluated changes in search activity during the COVID-19 pandemic period, relevant cancer awareness campaigns, and cancer-related events receiving major publicity, and analysed the content of related searches qualitatively to determine the extent to which online information-seeking behaviour reflects public health priorities in cancer prevention and early detection. The main hypotheses of our study were as follows: (i) online search activity would only partially correspond to the epidemiological burden of the selected cancers, because search behaviour is shaped not only by disease frequency and mortality, but also by public awareness, media visibility, perceived threat, available screening options and health literacy; (ii) institutional cancer awareness campaigns would be associated with limited or heterogeneous changes in search intensity; (iii) highly publicized cancer-related events involving well-known public figures would generate more pronounced short-term increases in online interest. Accordingly, our study was designed to examine not only long-term search trends, but also the extent to which digital public interest aligns with epidemiological priorities, screening-related communication needs and media-driven attention.

## 2. Materials and Methods

### 2.1. Study Design

A retrospective, observational, infodemiological study was conducted to analyse online information-seeking behaviour of the Hungarian public related to major cancer types of public health importance (i.e., lung, breast, colorectal, prostate and pancreatic cancer) using GT, corresponding to the period between 1 January 2013 and 31 December 2022. The 10-year study period allowed for carrying out longitudinal analyses, and to assess potential changes occurring the period affected by the COVID-19 pandemic. Raw data were collected between 19 February 2024 and 3 March 2024. Hungary was the primary setting, while global search trends from GT data were used for international contextual comparisons.

### 2.2. GT Data Collection

GT was selected as the primary data source because Google^TM^ maintained a dominant share of the global search engine market (both on personal computers and mobile devices) throughout the study period, thereby providing broad coverage of population-level online search activity [28]. GT provides anonymized, aggregated indices of search intensity as relative search volume (RSV) values. RSV is a normalized ratio ranging from 0 to 100, where 100 represents the peak search interest for a given search term or keyword in the specified time and region, while 0 indicates the lowest observed search intensity [26,41]. To improve the internal validity of the method, we focused on regions with high and increasing internet coverage; according to the Hungarian Central Statistical Office (HCSO), the percentage of Internet use among the Hungarian population aged 16–74 years increased from 73.0% of the population in 2013 to 89.0% in 2022, while World Bank data indicate that the proportion of individuals using the Internet globally increased from 36.0% to 63.0% over the same period [42,43]. According to previous methodological recommendations for country-level GT-analyses [27,34,35], the starting year of our study was set at 2013, when the proportion of Internet users in Hungary was deemed to be adequate (≥70%) to capture population-based information-seeking on the topic.

Uncorrected RSV values (RSV_raw_) were used as the primary indicator of search intensity in the main analyses based on GT data. As GT RSV values are already normalized relative measures of search activity within a selected time period, and correspond to a specific geographical setting, they should not be interpreted as absolute search counts or population-based search rates. As a part of our study, to explore whether the secular increase in Internet users over the study period could have influenced long-term trends, we additionally performed an Internet penetration-adjusted sensitivity analysis. For this sensitivity analysis, year-specific adjustment factors were calculated by dividing the proportion of Internet users in each year by the corresponding proportion in 2013 (used as reference), and RSV_raw_ values were divided by these adjustment factors. The resulting investigator-derived values were referred to as RSV_sens_ and should be interpreted only as sensitivity estimates, rather than as validated population-adjusted search rates. The percentage of Internet users and the corresponding sensitivity adjustment factors used for the Hungarian and worldwide analyses are provided in Supplementary Material S1. For descriptive, qualitative comparisons across different search terms and keywords, reference-adjusted RSV (RSV_ref_) values were additionally calculated, by expressing search intensity relative to a selected reference search term. As this procedure may yield values exceeding 100, RSV_ref_ values were used exclusively for descriptive comparisons and were not included in statistical analyses.

**Figure 1:**
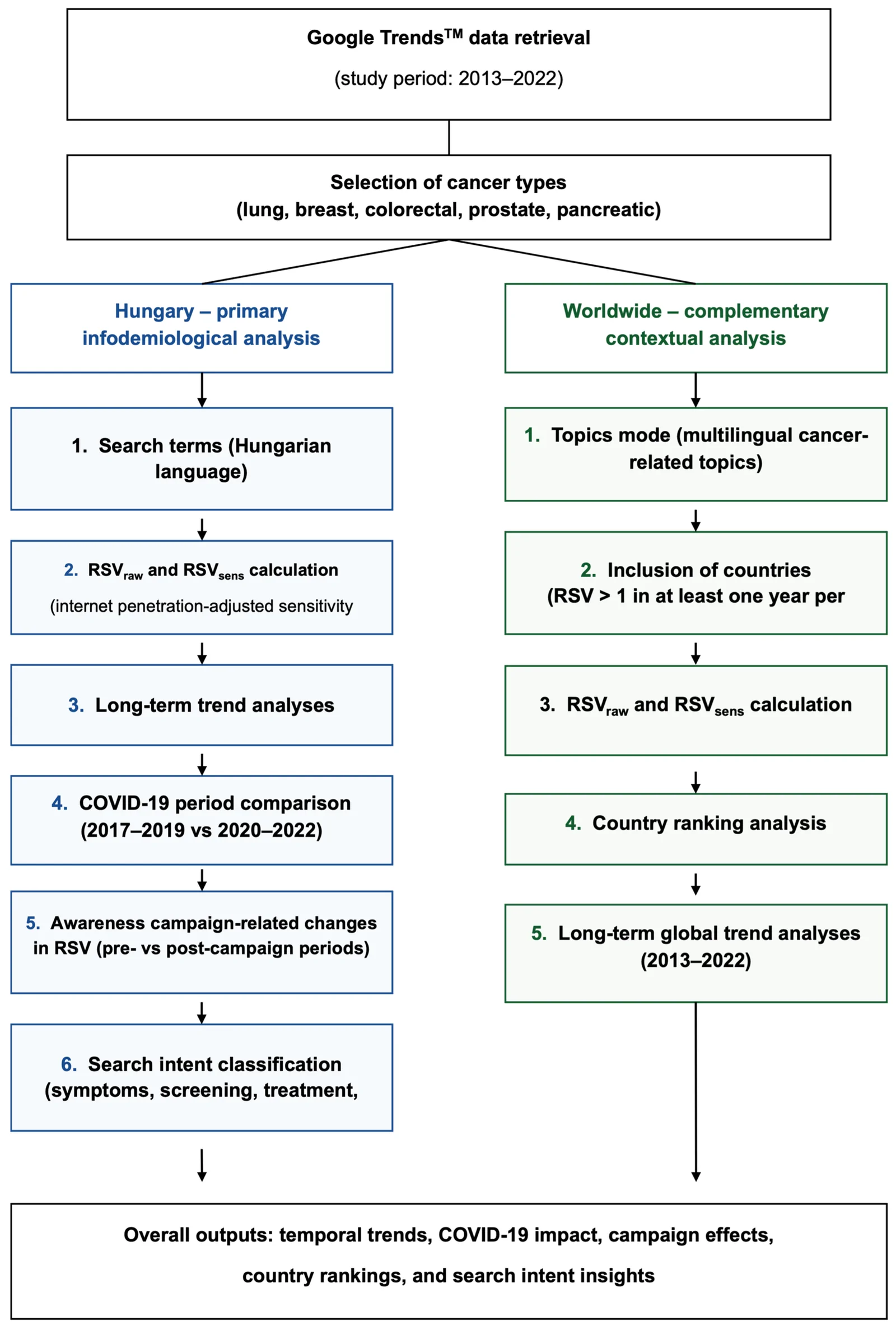
Workflow summarizing the Google Trends^TM^-based infodemiology study in Hungary, corresponding to major cancer types, and its complementary global contextual analysis; RSV: relative search volume; RSV_raw_: raw (uncorrected) relative search volume; RSV_sens_: investigator-derived RSV values used in the Internet penetration-adjusted sensitivity analysis.

While the platform is neutral with regard to disputes on borders and jurisdictions, when using the “Worldwide” geographical designation in GT for data collection, RSV data may be collected from more than 250 countries and regions. For global comparisons and country rankings, we primarily included countries with measurable RSVs (≥1) for the respective cancer topic. Non-measurable RSV values were interpreted as search activity below the GT reporting threshold, and were assigned a value of 0, only to be used in the exploratory analysis including all countries and territories. A graphical workflow diagram (Figure 1) summarizes the main steps of the analysis, including GT data extraction, calculation of RSV_raw_ and RSV_sens_ values, trend analyses, assessment of changes during the COVID-19 period, and awareness campaign-associated changes, country ranking analysis and classification of related queries by perceived information-seeking intent. As indicated by notes displayed in GT, the platform algorithm was updated on 1 January 2016 and 1 January 2022, respectively. For both the Hungarian and worldwide analyses, RSV data were retrieved using the “Web Search” option.

In addition to GT data, Human Development Index (HDI) values for 2022 for countries and territories were obtained from the United Nations Development Programme (UNDP) Human Development Data Center; HDI represents a composite measure of life expectancy at birth, expected and mean years of schooling, and gross national income per capita adjusted for purchasing power parity [44].

### 2.3. Selection of Cancer Types and Search Queries

The present infodemiological analysis focused on cancer types of major public health importance, i.e., tracheal, bronchial, and lung cancer (hereafter referred to as lung cancer); colon and rectal cancer (hereafter referred to as colorectal cancer); breast cancer; prostate cancer; and pancreatic cancer. For Hungary, RSV data were retrieved using the “Search term” function in GT, which returns search intensity for specific Hungarian keywords used verbatim only (e.g., „mellrák” for breast cancer). As a first step, we identified the Hungarian search terms with the highest overall search intensity related to the selected cancers (taking into account which would be used most commonly by the general population), namely “tüdőrák” (lung cancer), “mellrák” (breast cancer), “vastagbélrák” (colorectal cancer), “prosztatarák” (prostate cancer) and “hasnyálmirigyrák” (pancreatic cancer), respectively. As a comparator and reference for our final selection of search terms, the search intentisty for some non-cancer related search queries were also assessed.

For the analysis of global search intensity, the geographical location assigned was “Worldwide”, and RSV_raw_ values were collected using the “Topic” function in GT, which aggregates conceptually related queries across languages and synonyms, and is therefore more suitable for international comparisons. This approach may have introduced a methodological asymmetry between the Hungarian and global analyses: in case of Hungary, the use of the “Search Term” function allowed us to capture the exact Hungarian keywords most likely to be used by the general population; however, it does not include synonyms, spelling variants, or related derivative forms (although our exploratory analyses of relevant keywords aimed to reduce the probability of introducing bias). By contrast, the “Topic” function used for global comparisons aggregates conceptually related queries across languages and synonyms. Therefore, Hungarian “Search Term”-based RSV values may underestimate broader cancer-related search interest compared with “Topic”-based global RSV values. For this reason, international comparisons were interpreted as contextual and exploratory rather than as direct one-to-one comparisons of absolute search interest. The corresponding topics were “breast cancer”, “lung cancer”, “prostate cancer”, “colorectal cancer” and “pancreatic cancer”, respectively, for the same time-frame as the Hungarian data collection.

### 2.4. Changes During the COVID-19 Pandemic, Public Health Educational Campaigns and Other Events Involving Public Figures

To assess changes in cancer-related search intensity associated with the COVID-19 pandemic, we defined a pre-pandemic baseline period (2017–2019) and a period affected by the pandemic (i.e., “COVID-19 period”; 2020–2022). Mean RSV values and 95% confidence intervals (95% CI) were calculated for each period, and absolute and relative changes in mean RSV between the two periods were evaluated for each cancer type. To assess changes in search intensity associated with cancer-related awareness campaigns, we constructed predefined time windows around the World Cancer Day (4 February each year), and cancer-specific campaigns. For World Cancer Day, mean weekly RSV values and 95% CIs were calculated for the week preceding the campaign and for a four-week post-event window, consisting of the campaign week and the subsequent three weeks. This window was selected to capture both immediate and delayed search responses to a short public health awareness event, while remaining comparable to the monthly time frames commonly used in GT studies evaluating health awareness events and public health campaigns [45]. Changes associated with cancer-specific awareness campaigns were evaluated by comparing mean RSV values during the calendar month immediately preceding each campaign with mean RSV values during the respective awareness month. The predefined awareness periods were October for breast cancer (“Breast Cancer Awareness Month/Pink October”), March for colorectal cancer (“Colorectal Cancer Awareness Month”), and November for lung (“Lung Cancer Awareness Month”), prostate (“Prostate Cancer Awareness Month/Movember”), and pancreatic cancer (“Pancreatic Cancer Awareness Month”), respectively. Accordingly, September was compared with October for breast cancer, February with March for colorectal cancer, and October with November for lung, prostate, and pancreatic cancer, respectively.

Media events related to pancreatic cancer were identified based on published reports concerning highly publicized cases involving prominent Hungarian figures, including the public disclosure of Péter Esterházy (P.E.)’s diagnosis (October 2015), his subsequent death (14 July 2016), and the death of Tibor Benedek (T.B.) (18 June 2020). For the P.E.-related events, pre- and post-event mean RSV values were calculated over predefined four-week periods.

### 2.5. Search Intent Analysis

To characterize cancer-related information-seeking behaviour, we retrieved the most frequent GT “related queries” for each cancer type over the study period (2013–2022), in the form of a qualitative analysis of search terms. Related queries were collected yearly, using the “Top” function for their classification, and queries that appeared repeatedly across multiple years were retained to assess temporal consistency. All identified queries were independently reviewed and classified according to their primary information-seeking intent, using a study-specific classification framework developed by the research team. The framework comprised five categories: (1) symptoms; (2) screening and early detection of disease; (3) treatment; (4) disease prognosis; and (5) other cancer-related information (including benign conditions, patient support, and non-evidence-based explanations). Two researchers with experience in public health and cancer epidemiology (M.G. and Zs.K.) independently assigned each query to one category. Inter-observer agreement between the two independent reviewers was assessed before consensus resolution using Cohen’s κ statistic [46]. Disagreements were resolved through discussion and, when necessary, by consultation with a third senior researcher (E.P.) until consensus was reached. The final consensus-based categories were used in the descriptive search intent analysis.

### 2.6. Statistical Analysis

Following data extraction, raw data were downloaded from the GT interface in comma-separated values (.csv) format, using the predefined settings and filters described above, then imported into Microsoft Excel 365 (Redmond, WA, USA) for data management and initial cleaning, sorting and graphical representation. For descriptive statistics, inferential analyses, and time series trend analyses, data were imported into IBM SPSS Statistics 22.0 (Armonk, NY, USA) and Paleontological Statistics Software Package (PAST) 4.03 (University of Oslo, Norway), while Joinpoint version 5.3.0 (National Cancer Institute, Bethesda, MD, USA) was used for joinpoint regression analyses, where average annual percent changes (AAPCs) and corresponding 95% CIs were estimated.

Descriptive statistics are presented as means with standard deviations (SDs) or 95% CIs, as appropriate. Absolute and relative (%) changes in RSV values were calculated to quantify differences between comparison periods. Long-term temporal trends in RSV values were assessed using linear regression, with the slope coefficient (β) serving as a descriptive indicator of the direction and magnitude of change. The main trend analyses were based on RSV_raw_ values. RSV_sens_ values were analysed as an Internet penetration-adjusted sensitivity analysis, to explore whether long-term patterns were consistent after accounting for secular changes in Internet use rate. The statistical significance of monotonic trends was evaluated with the Mann–Kendall (MK) test, and the Wald–Wolfowitz (WWR) runs test was applied to examine whether the temporal ordering of observations deviated from randomness. Correlation analysis between country-level search intensity data and HDI values, and between search intensity data among different cancers of public health importance, was performed using Pearson’s correlation coefficient (r_p_); the relationship between the variables was classified as follows: |r_p_| < 0.3, weak; 0.3 ≤ |r_p_| < 0.5, moderate; 0.5 ≤ |r_p_| < 0.8, strong; and |r_p_| ≥ 0.8, very strong.

Differences between the pre-COVID-19 period and the COVID-19 period, as well as between pre- and post-campaign intervals, were evaluated using parametric tests. In line with previous infodemiological studies [27], RSV values were treated as approximately normally distributed, continuous variables; paired-samples t-tests were used for comparisons between the pre-COVID-19 and COVID-19 periods and between paired pre- and post-campaign intervals. One-way analysis of variance (ANOVA) and Tukey’s post hoc tests were applied where comparisons involved multiple independent groups. Mean RSV values, absolute and relative percentage changes and *p*-values are reported. Weekly RSV values were first extracted, and then aggregated by calendar month to explore potential seasonality in cancer-related search activity. Seasonal variation in monthly RSV distributions was assessed using the Kruskal–Wallis H test. Statistical significance throughout our analyses was defined as *p* < 0.05. Given the number of campaign- and cancer-specific pairwise comparisons and multiple correlation matrices, a Benjamini–Hochberg (BH) false discovery rate (FDR) correction was applied to reduce the probability of Type I error, due to multiple testing; BH is considered appropriate for exploratory analyses. In the Results Section, BH-adjusted *p*-values (*q*-values) are reported, with *q* < 0.05 considered as the threshold for significance.

### 2.7. Reporting Guidelines

The study was reported in accordance with the STROBE (Strengthening the Reporting of Observational Studies in Epidemiology) statement [47] and followed methodological recommendations for GT-based health research as proposed by Mavragani and colleagues [27]; the STROBE checklist is provided in Supplementary Material S2.

## 3. Results

### 3.1. Overall Search Interest Across Cancer Types

**Figure 2:**
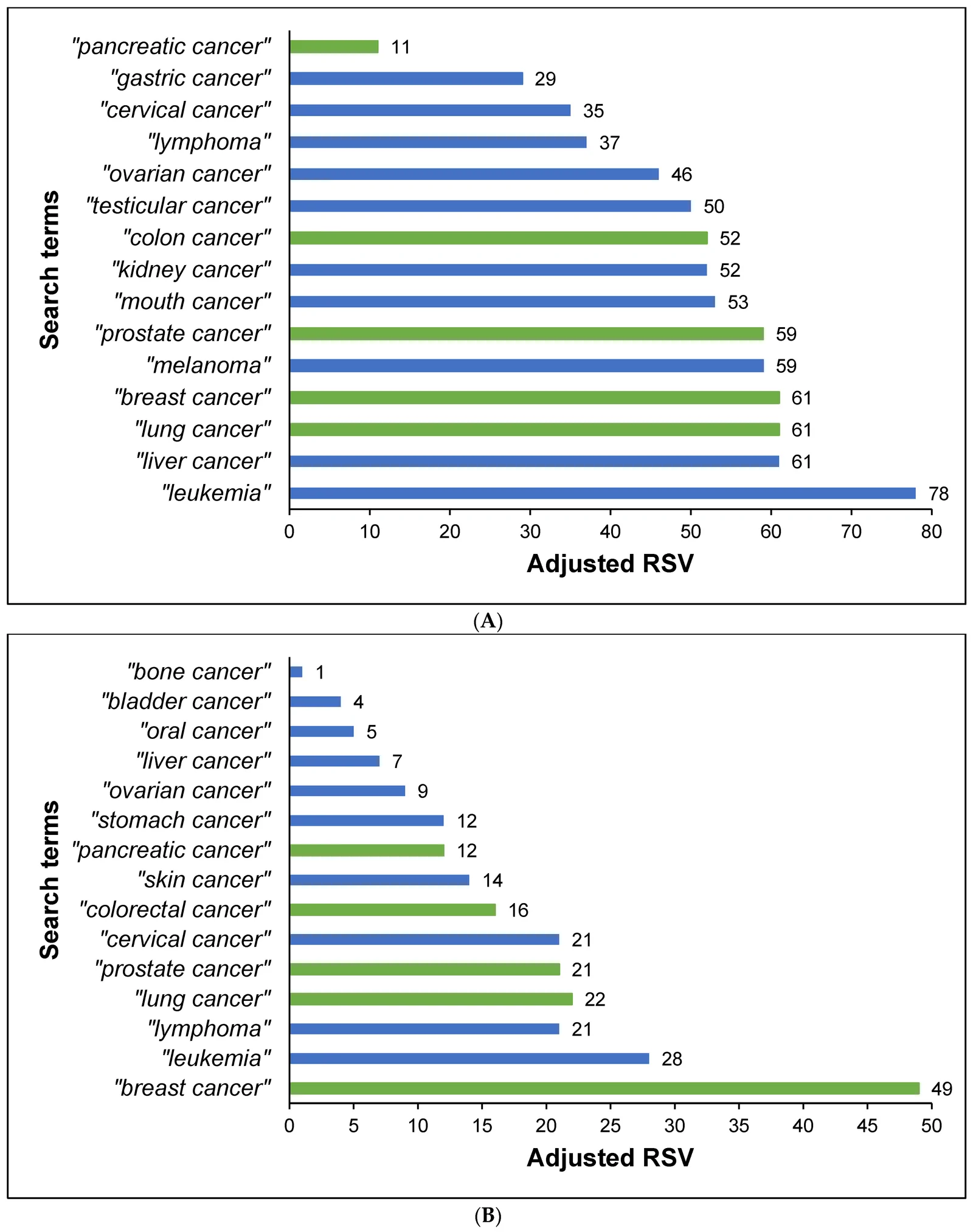
(**A**) Ranking of major cancer-related search terms in Hungary over the study period (2013–2022), by reference-adjusted relative search volume (RSV_ref_); Hungarian data are based on GT “search term” queries; numeric labels at the end of each bar indicate the corresponding adjusted RSV values for the 10-year period. Light green bars highlight the five cancer types included in the main analyses (breast, lung, colorectal, prostate and pancreatic cancer), whereas dark blue bars indicate search terms for unrelated cancer types. Horizontal bars show the mean reference-adjusted relative search volume (RSV_ref_) for cancer-related search terms; absolute RSV values are not directly comparable with global topic-based data, and the analysis focuses on relative patterns of search interest across cancer types. (**B**) Ranking of major cancer-related topics worldwide over the study period (2013–2022), by reference-adjusted relative search volume (RSV_ref_). Worldwide data are based on GT “topic” queries for the 10-year period; numeric labels at the end of each bar indicate the corresponding adjusted RSV values. Light green bars highlight the five cancer types included in the main analyses (breast, lung, colorectal, prostate and pancreatic cancer), whereas dark blue bars indicate search terms for unrelated cancer types. Horizontal bars show the mean reference-adjusted relative search volume (RSV_ref_) for cancer-related search terms; absolute RSV values are not directly comparable with global topic-based data, and the analysis focuses on relative patterns of search interest across cancer types.

The comparative online search intensity of the major cancer types included in the study showed notable differences between Hungary and the global context. In Hungary, among the major cancer types included in our study, lung and breast cancer showed the highest relative search interest (RSV*_ref_*: 61 in both cases), followed by prostate (RSV*_ref_*: 59), colorectal (RSV*_ref_*: 52), and pancreatic cancer (RSV*_ref_*: 11) (Figure 2A); however, comparatively, other cancer types—such as leukemia (RSV*_ref_*: 78), liver cancer (RSV*_ref_*: 61) and melanoma (RSV*_ref_*: 59) also presented with notable search intensity.

Compared to lung cancer search intensity in Hungary, search intensity for the reference search term asthma (“asztma”) was comparable (RSV*_ref_*: 61) within the same time frame, while smoking (“dohányzás”; RSV*_ref_*: 107), pneumonia (“tüdőgyulladás”; RSV*_ref_*: 136) and a colloquial term for cigarettes (“cigi”; RSV*_ref_*: 412) showed notably higher search intensity. Compared to breast cancer, lactation (“szoptatás”; RSV*_ref_*: 190) was considerably higher, while other reference terms, such as breast augmentation („mellplasztika”; RSV*_ref_*: 54), breast surgery (“mellműtét”; RSV*_ref_*: 18) and breast implant (“mell implantátum”; RSV*_ref_*: 2) were notably less relevant. In the context of prostate cancer, prostatitis (“prosztatagyulladás”; RSV*_ref_*: 75) showed higher, while prostate enlargement (“prosztata megnagyobbodás”; RSV*_ref_*: 24) had a lower search intensity score than the cancer-related search term during the study period. In relation to colorectal cancer, the search term bloody stool (“véres széklet”; RSV*_ref_*: 48) had comparable search intensity, while colorectal polyp (“vastagbél polip”; RSV*_ref_*: 8) showed considerably lower search intensity; in addition other relevant terms, such as constipation (“székrekedés”; RSV*_ref_*: 119), the colloquial term for hemorrhoid (“aranyér”; RSV*_ref_*: 426) and diarrhea (“hasmenés”; RSV*_ref_*: 542) had many-fold higher search intensities than the cancer-related term. Finally, when compared to pancreatic cancer, the search terms for diabetes (“cukorbetegség”; RSV*_ref_*: 77), and pancreatitis (“hasnyálmirigy gyulladás” RSV*_ref_: *26) carried notably higher cumulative search intensity.

In contrast, in the global context, breast cancer had the highest search intensity (RSV*_ref_*: 49), while prostate (RSV*_ref_*: 21) and lung cancer (RSV*_ref_*: 22) elicited similar levels of relative interest, followed by colorectal (RSV*_ref_*: 16) and pancreatic cancer (RSV*_ref_*: 12) (Figure 2B). As in Hungary, blood cancers (leukemia RSV*_ref_*: 28, lymphoma: RSV*_ref_*: 21) also generated substantial online interest, when compared to other cancer types. Although the relative ranking of cancer types showed substantial differences between settings, breast, lung and prostate cancer consistently belonged to the group of cancers that generated the greatest online interest both in Hungary and globally, whereas pancreatic cancer ranked among the cancer types characterized by lower search intensity in both contexts.

In the global country rankings—according to RSV values—Hungary’s position differed markedly across cancer types. For searches related to pancreatic cancer, Hungary ranked 29th of 60 countries (RSV = 31) with measurable search intensity (RSV ≥ 1), while for lung cancer, it ranked 30th of 50 countries (RSV = 45). In contrast, Hungary occupied much lower positions for colorectal cancer (63rd of 67 countries; RSV = 19), prostate cancer (66th of 72 countries; RSV = 16) and breast cancer (72nd of 74 countries; RSV = 18), placing the country in the lower part of the global rankings—among countries with measurable search intensity—for these cancer types (Table 1), respectively. The international patterns showed distinct geographic concentrations across cancer types. For lung and colorectal cancer, several East Asian and English-speaking countries appeared among the top-ranked positions, whereas the highest relative interest in prostate cancer was observed primarily in Latin American and African countries. In contrast, the country distribution of breast and pancreatic cancer-related searches was more geographically diverse (Supplementary Material S3).

As a part of our study, the relationship between country-level RSV-values—corresponding to each cancer type of interest—and HDI values were assessed: when only countries with measurable search intensity (RSV ≥ 1) were included in our analysis, significant positive correlations were noted between HDI values and colorectal cancer (r = 0.405) and pancreatic cancer (r = 0.596), while negative correlation was shown for prostate cancer (r = −0.274). On the other hand, if all countries and territories were included in our analysis (in this case, RSV values were considered as 0, if they did not satisfy our earlier criteria), significant correlations were noted between HDI and country-level search intensity for all studied cancer types: in the case of pancreatic, colorectal and lung cancer, the direction of this correlation was positive (higher HDI values were associated with higher search intensity), while in the context of prostate and breast cancer, an inverse correlation was shown (Table 2). With the exception of breast cancer in the second analysis, all results retained significance after BH-correction.

Additionally, the correlations between the search intensities for relevant cancer types in the Hungarian context were also determined. With the exception of pancreatic cancer, for which no significant associations were noted with RSVs for other cancer types, significant positive correlations were observed in several comparisons between lung, colorectal, prostate and breast cancer-associated search intensities (Table 3). This suggests that pancreatic cancer-related search activity followed a distinct temporal pattern compared with the other major cancers examined. Rather than moving in parallel with broader cancer-related search interest, pancreatic cancer search intensity appeared to be more strongly influenced by isolated short-term peaks, which would later be identified to coincide with highly publicized cancer-related events involving well-known public figures.

### 3.2. Long-Term Temporal Trends in Cancer-Related Search Intensity in Hungary and Globally (2013–2022)

The temporal analysis of cancer-related search intensity in Hungary showed decreasing linear trends (based on *β*) for all examined cancer types based on RSV_raw_ values. In the Internet penetration-adjusted sensitivity analysis (RSV_sens_), decreasing linear trends were also observed for all cancer types. Based on the MK test, significant monotonic decreasing trends were shown for RSV*_raw_* for lung, breast, colorectal and pancreatic cancer, while this decreasing tendency for prostate cancer was not significant. In contrast, the analysis of RSV_sens_ values demonstrated a significant decreasing monotonic trend for all cancer types in our analysis. Based on the average annual percent change (AAPC), the largest yearly decreases were observed for pancreatic cancer (−6.97%) and colorectal cancer (−6.59%), while search intensity for lung cancer fell by an average of 4.14% per year. Smaller annual decreases were detected for breast and prostate cancer, and the corresponding AAPC estimates for raw RSV were not statistically significant. In the RSV_sens_ sensitivity analysis, significant yearly decreases were observed for all cancer types included in the study (Table 4). Further, the WWR test indicated non-random temporal patterns for lung, breast, prostate and pancreatic cancer-related searches, whereas colorectal cancer showed insufficient evidence (*p* > 0.05) of deviation from randomness (Table 4).

Globally, long-term trends partially differed from the Hungarian patterns. Based on raw RSV data, no clear monotonic trends were observed for lung, prostate and breast cancer (*p* > 0.05), whereas colorectal and pancreatic cancer showed increasing search intensity. When RSV_sens_ values were analysed, significant long-term decreasing trends observed for all examined cancer types, associated with the linear regression, Mann–Kendall and runs-test analyses (Supplementary Material S4).

**Figure 3:**
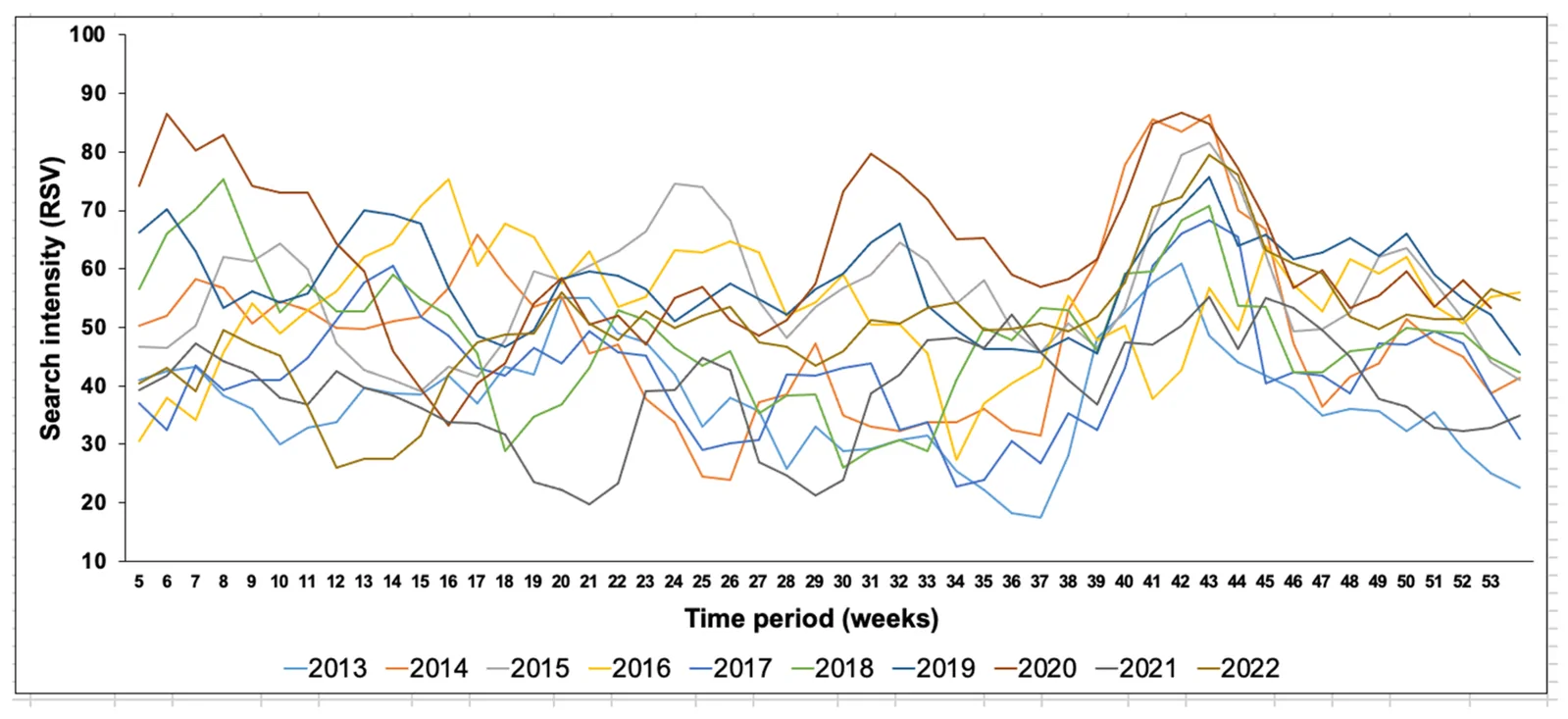
Four-week moving average of weekly relative search volume (RSV_raw_) for breast cancer (“mellrák”) in Hungary, 2013–2022; the lines represent four-week moving averages of weekly RSV values for each year of the study period; the x-axis represents calendar weeks.

**Figure 4:**
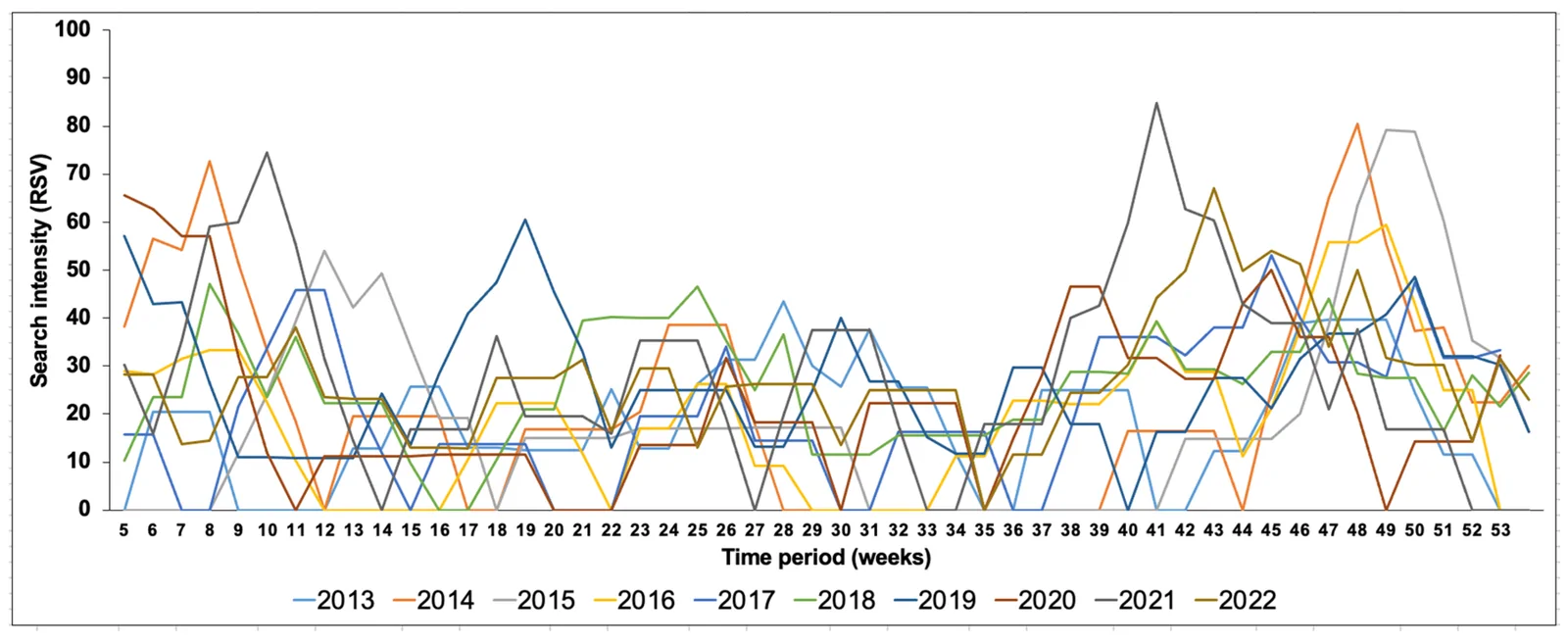
Four-week moving average of weekly relative search volume (RSV_raw_) for prostate cancer (“prosztatarák”) in Hungary, 2013–2022; the lines represent four-week moving averages of weekly RSV values for each year of the study period; the x-axis represents calendar weeks.

A more granular analysis of seasonality was performed separately by analyzing weekly RSV values across the calendar year and summarising the presence or absence of seasonal variation (Table 4). This analysis revealed significant seasonal variation for breast and prostate cancer, with consistently higher search intensity during weeks 40–46 and 37–52, respectively, while no significant seasonal variation was observed for lung, colorectal or pancreatic cancers. However, these weekly seasonal patterns should not be interpreted as direct evidence of a statistically significant campaign-associated monthly effect. Although breast and prostate cancer showed significant seasonality, when assessed on a weekly scale, the magnitude of the seasonal increase was not sufficiently uniform across individual years to produce a statistically significant increase in the paired monthly comparison of the awareness month with the preceding month. The seasonal patterns for breast and prostate cancer are illustrated by the four-week moving averages of weekly RSV_raw_ values in Figure 3 and Figure 4.

### 3.3. Temporal Trends and Changes During the COVID-19–Related Period in Hungary

Cancer-related online search interest decreased during the COVID-19 period for all examined cancer types in Hungary; the decline was statistically significant for lung, breast, prostate and colorectal cancer, while this was not the case for pancreatic cancer (Table 5). The largest absolute and relative decrease was observed for lung cancer (ΔRSV: −20.33; Δ%: −30.2%), while colorectal cancer showed the smallest, but still statistically significant reduction in search intensity (resulting in a *q*-value of 0.053 following BH-correction). Although pancreatic cancer also showed a numerical decline during the COVID-19 period (Δ%: −16.5%), this change did not reach statistical significance. This may be explained by the higher variability of pancreatic cancer-related search intensity, which was influenced by isolated short-term spikes associated with highly publicized events involving well-known public figures.

### 3.4. Campaign Responsiveness by Cancer Type

Our study assessed the potential influence of general and cancer-specific educational awareness campaigns on online search intensity, within the Hungarian context. Following the World Cancer Day, only modest changes in online search intensity were observed across cancer types, which did not substantially change throughout the three-week latency period set for the comparison. Mean RSV increased slightly for lung (Δ%: +5.9%) and breast cancer (Δ%: +9.0%), while prostate (Δ%: −4.2%), colorectal (Δ%: −3.2%) and pancreatic cancer (Δ%: −52.8%) showed decreasing RSV values; however, none of these changes reached statistical significance (*p* and *q* values > 0.05). On the other hand, the analysis of cancer-specific awareness campaigns revealed more heterogeneous patterns: search intensity values increased during the campaign periods for all examined cancers, but the magnitude of change varied considerably according to cancer type. The largest absolute (ΔRSV: +17.77) and relative (Δ%: +66.9%) increase was observed for colorectal cancer, representing the only statistically significant campaign-associated change (*p* = 0.013; however, following BH-correction, the *q*-value was 0.058), whereas increases for breast (Δ%: +14.6%), pancreatic (Δ%: +56.8%), prostate (Δ%: +2.2%) and lung cancer (Δ%: +0.5%) did not reach statistical significance (Table 6). Thus, the significant weekly seasonality observed for breast and prostate cancer did not translate into a statistically significant paired monthly campaign-associated increase, suggesting that the observed seasonal peaks varied in magnitude across years.

### 3.5. Publicized Diagnoses and Deaths of Public Figures, and Spikes in Pancreatic Cancer-Related Search Intensity

**Figure 5:**
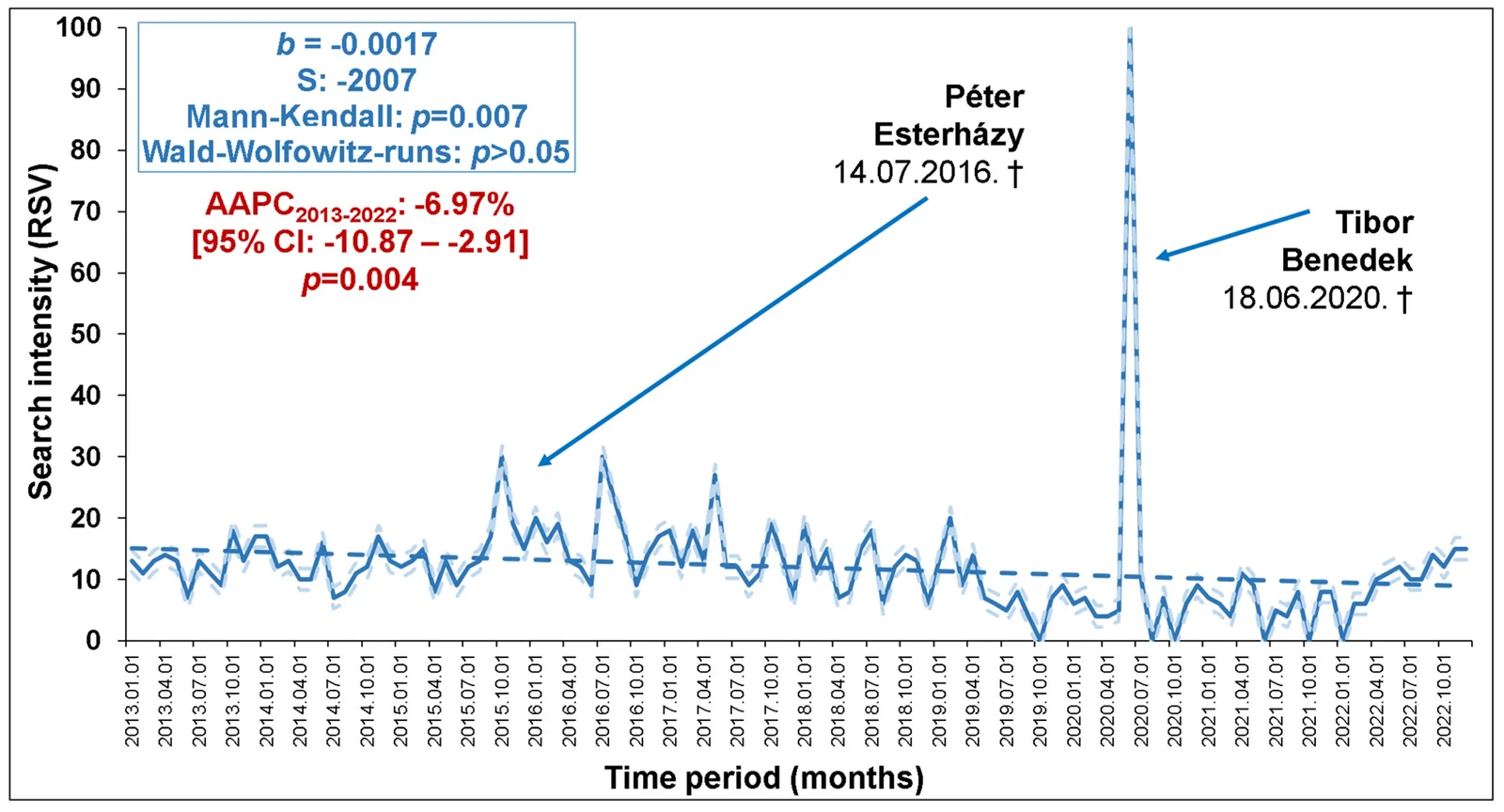
Monthly pancreatic cancer-related search intensity in Hungary based on uncorrected RSV, 2013–2022, with long-term trend and media events. Note: RSV: relative search volume; β: linear regression slope; S: Mann–Kendall test statistic; AAPC: average annual percent change; CI: confidence interval. The solid line represents monthly relative search volume (RSV_raw_) for pancreatic cancer in Hungary during the study period, and the dashed line represents the fitted linear trend. The panel reports the linear trend slope (β), Mann–Kendall S and *p*-value, Wald–Wolfowitz runs test *p*-value, and average annual percent change (AAPC) with its 95% confidence interval. Arrows indicate the timing of the deaths of Péter Esterházy (14 July 2016) and Tibor Benedek (18 June 2020). The sing “†” corresponds to the time of death.

Consistent with its distinct correlation pattern, two highly publicized cases of pancreatic cancer were associated with marked short-term increases in online search intensity in Hungary. In the case of Péter Esterházy (P.E.), mean RSV for pancreatic cancer was 7.16 (95% CI: 0.45–11.86) in the four-week period preceding the public disclosure of his diagnosis in October 2015, and increased to 28.86 (95% CI: 12.81–44.89) in the subsequent four weeks, representing a marked and statistically significant increase in search intensity (*p* < 0.001; *q* < 0.001). Subsequently, in the weeks surrounding his death in July 2016, mean RSV rose again from 8.00 (95% CI: 1.28–14.72) before the event to 35.72 (95% CI: 25.78–49.86) afterwards, corresponding to another statistically significant increase (*p* < 0.001; *q* < 0.001). In the case of Tibor Benedek (T.B.), mean RSV for pancreatic cancer in the four-week period preceding his death was 0.71 (95% CI: −1.03–2.46), and increased to 26.17 (95% CI: −15.43–67.76) in the subsequent four weeks, representing a marked and statistically significant increase in search intensity (*p* < 0.001; *q* < 0.001). In relative terms, mean RSV increased approximately four-fold after the public disclosure of P.E.’s diagnosis and more than four-fold after his death. The largest relative change was observed after the death of T.B., when mean RSV increased from a very low pre-event level of <1 RSV to over 26 RSV points, respectively. As this increase originated from a very low baseline, it should be interpreted cautiously; nevertheless, these public figure-related peaks represented the most pronounced short-term deviations in pancreatic cancer-related search intensity during the study period. These peaks in search intensity (two smaller peaks for P.E. in 2015 and 2016, while one major peak in 2020, corresponding to T.B.) can be clearly identified in Figure 5.

### 3.6. Search Intent Patterns in Cancer-Related Queries

The analysis of related search terms showed that online information-seeking related to cancer was dominated by queries targeting information on symptoms across all examined cancer types; results are summarized in Table 7, while detailed term-level RSV information is presented in Supplementary Material S5. Inter-observer agreement for the independent classification of the retained related queries was excellent, with a Cohen’s κ value of 0.924. The two reviewers agreed on 77 of 81 queries (95.1%); disagreements were resolved through consensus and, when necessary, by consultation with a third senior reviewer. The terms “lung cancer symptoms”, “breast cancer symptoms”, “colorectal cancer symptoms” and “prostate cancer symptoms” consistently reached the highest relative search volumes (RSV: 100) in all study years, and were present throughout the entire study period. For pancreatic cancer, the query “pancreatic cancer symptoms” also showed persistently high RSV values (ranging from 86 to 100 in the years in which it appeared among the top related queries), while the more general “pancreas” query reached comparable or even higher RSV values (ranging from 50 to 100) in a number of years.

Searches related to screening and early detection were much more limited. Screening-related queries were identified for breast, colorectal and prostate cancer, but their temporal patterns and intensity differed markedly. The term “colorectal cancer screening” appeared throughout the study period with modest RSV values (ranging from 17 to 34), indicating a relatively stable interest in this screening modality. In contrast, breast cancer screening queries were essentially confined to the beginning of the period (with low RSVs around 6), and prostate cancer screening queries were present only in several consecutive years (RSV ranging from 6 to 30) before disappearing from the leading searches after 2018. For lung and pancreatic cancer, no prominent screening- or early detection-related terms were identified among the top related queries. Overall, these findings indicate a marked quantitative imbalance between reactive, symptom-oriented information seeking and proactive interest in screening or early detection of cancers. While symptom-related queries consistently reached the maximum RSV value across the examined cancer types, screening-related queries were either absent from the leading related searches, or appeared with substantially lower RSV values, ranging from 6 to 34 (vs. 100). This pattern was particularly evident for prostate cancer, where screening-related queries were observed only up to 2018, and did not remain among the leading related searches thereafter.

Beyond symptoms and screening, related searches covered other topics, such as treatment (e.g., “lung cancer treatment”, “prostate cancer cure”), and disease prognosis (e.g., “lung cancer stages”, “colorectal cancer survival”), although these categories generally showed lower and less consistent search intensities. In addition, several cancers were associated with queries referring to presumed “psychological causes” of the disease (e.g., “breast cancer psychological causes”, “colorectal cancer psychological causes”, “pancreatic cancer psychological causes”), representing other cancer-related information and pointing to public interest in non-evidence-based explanations of cancer, even though the intensity and timing of such searches fluctuated over time.

## 4. Discussion

Malignant disorders are increasingly consequential illnesses, with 1 in 9 men and 1 in 12 women currently expected to die from cancer, and the cancer-related disease burden is projected to increase—especially in LMICs—principally due to demographic changes, and the greater emergence of some lifestyle-related risk factors. While there is substantial geographical heterogeneity in the relevance of certain cancer types, others (such as lung, breast and colorectal cancer) have a notable disease burden throughout the globe [1,3]. Given the importance of this topic, 4 February was designated as “World Cancer Day” at the World Summit Against Cancer for the New Millennium in 2000, held in Paris [48]. In addition, a plethora of dedicated educational campaigns have also been established for various subtypes of cancers (e.g., Breast Cancer Awareness Month or “Pink October”), which often originated from or were taken up by healthcare organizations and patient advocacy groups as well [49].

Health-related information-seeking from various online sources and the ”digitally informed” patient are increasingly becoming the norm in many countries, especially since the COVID-19 pandemic [50,51]; while there are certain advantages to this phenomenon (i.e., increased agency and empowerment, improved access to health information, support for informed decision-making, more realistic expectations towards the outcomes of medical treatment, and a greater tendency to participate in shared decision-making in the context of medical decisions), there are also considerable risks, including the availability of low-quality, non-evidence-based or outright misleading health information, which may result in adverse outcomes, inappropriate value judgements, aversion to participating in certain screening or treatment modalities or cancer fatalism [50,52]. At the same time, healthcare professionals must also be upskilled with the relevant competencies, to be effective and proactive partners for “digitally informed” patients, including the ability to address the phenomenon of “Dr Google” and to help patients identify and debunk health misinformation [52,53,54].

Within the context of EU_27_ countries, the mean percentage of individuals using the Internet to seek health-related information (e.g., injury, disease, nutrition, improving health) within the last 3 months—among people aged 16 to 74 years—was 56.3% according to 2023 data; furthermore, Hungary was above the EU_27_ average, with 67.3% [55]. Additionally, a recent survey among 15-29-year-olds in Hungary highlighted that, in this age group, online sources are important information sources for health-related inquiries, with over two-thirds (68.0%) of respondents reporting search engines, while 23.0% reported social media sites as starting points for their enquiries [56]. Therefore, understanding patterns of public online health information seeking is becoming increasingly important, as digital platforms play a growing role in cancer awareness and health communication. Infodemiological approaches enable public health professionals to respond more dynamically to population concerns and behaviours shaped by the internet, helping bridge the gap created by reverse information asymmetry, and may rapidly provide information to relevant stakeholders to support health policy and public health decision-making [27,57].

Using ten years of search intensity data from GT, our study provides a longitudinal, comprehensive overview of online information seeking related to five major cancer types in Hungary, complemented by comparisons in an international context. To the best of our knowledge, this is the first Hungarian infodemiological investigation to simultaneously evaluate long-term search trends across multiple key cancers and to integrate temporal analyses, assessment of COVID-19-related changes, search patterns observed during public awareness campaign periods, and a qualitative assessment of search intent. Overall, our analyses yielded four main findings within the Hungarian context: (i) we observed a long-term decline in cancer-related search intensity for all cancer types included in our analysis; (ii) search intensity notably decreased for every cancer type during the period affected by the COVID-19 pandemic; (iii) marked changes in search activity did not occur at the same time as general cancer-awareness campaigns took place, whereas among dedicated campaigns for specific cancers, only those related to colorectal cancer were associated with a notable increase in search intensity; (iv) qualitative assessment of related queries indicated that public information seeking was clearly dominated by symptom-focused searches, while queries related to screening, prevention and early detection appeared much less frequently. Overall, these findings suggest that GT may be applied not only to monitor temporal changes in online interest regarding cancers, but also as a complementary tool to identify signals, such as population information needs, motivations for information seeking and potential gaps in cancer-related knowledge, which may be addressed via health communication.

One of the key observations of our study was the discrepancy between online search interest and local epidemiological realities and disease burden. Despite Hungary having one of the highest cancer mortality rates in Europe [3,5], with 25.1% of all deaths attributable to neoplasms in 2024 [58], breast, colorectal and prostate cancer showed relatively low search activity compared with other countries with measurable search intensity. When taking into account adjusted RSV_sens_ values, consistently decreasing intensity in cancer-related online information-seeking was shown for all major cancer types studied. In addition, pancreatic cancer served as an outlier among studied cancer types, in terms of its lack of significant correlation with the search intensity of other cancers. Previous methodological and review papers have emphasized that GT data primarily reflects population search interest and its temporal dynamics, and may only be used for epidemiological inference when appropriately validated against external data sources [27,29,36].

While cancer-related mortality in Hungary is highest for the cancers included in our analyses, other cancer types, i.e., blood cancers (lymphoma, leukemia), liver and kidney cancer, and cancers of the female reproductive system (ovarian, cervical) have received high rates of public interest comparatively, despite being considerably less common. However, participation in the Hungarian cervical cancer screening programme remains relatively low, declining from 47% in 2006 to 26% in 2021 [5]. Another possible explanation may be the media coverage of pediatric patients affected by blood cancers, including in the context of humanitarian and fundraising initiatives. This discrepancy illustrates that GT-based search intensity is strongly shaped by media salience and emotionally engaging narratives, rather than by disease burden in the region alone. Therefore, high online interest in a malignancy should not be interpreted as direct evidence of high population-level incidence or mortality (or extensive knowledge of the disease burden and preventive approaches), but rather as a signal of public attention that may be amplified by news coverage, personal stories and fundraising campaigns. Population interest is influenced by multiple factors—including media coverage, awareness campaigns, health literacy and cultural attitudes—highlighting that GT should be interpreted as a complementary indicator of public awareness, rather than a surrogate measure of cancer incidence or mortality [27,57].

Viewing the Hungarian results in an international comparison further reinforces this interpretation: although Hungarian and global data were obtained using different GT functionalities (i.e., “search terms” vs. “topics”), Hungary consistently ranked among the countries with lower search intensity for cancers, particularly for breast and colorectal cancer, despite the presence of organized screening programmes. Notably, nationwide organized mammography screening for breast cancer has been available in Hungary since 2002 [59]. This finding suggests that the opportunities for prevention and early detection may not be adequately reflected in public online information-seeking behaviour. Several explanations may account for this apparent paradox. Low online search intensity despite the availability of organised screening may reflect limited digital promotion of screening programmes, low perceived relevance of preventive cancer information, cancer fatalism, or the distrust toward healthcare services. However, GT data cannot distinguish between these mechanisms directly. Therefore, this finding should be interpreted as a signal of low spontaneous online information demand rather than as evidence of a specific underlying cause. Whether these patterns reflect differences in cancer-related beliefs, trust in healthcare or exposure to digital health communication requires further investigation. Similar studies have shown that Internet search activity often responds more strongly to media coverage and awareness campaigns than to underlying epidemiological trends [40,60]. Consequently, infodemiological approaches should be regarded not only as observational tools, but also as instruments for identifying communication gaps and supporting the development of more targeted cancer prevention strategies. The relationship between search intensity at the country level and HDI—acting as a summary measure for key dimensions of human development in a given country—also showed contrasting patterns across cancer types: while in the context of lung, colorectal and pancreatic cancer, higher search intensity was associated with higher HDI, the opposite pattern was observed for prostate and breast cancers. These divergent patterns suggest that socio-economic development may shape cancer-related information seeking differently across malignancies. Higher HDIs may increase online interest in cancers with stronger diagnostic, media or specialist-care visibility, whereas the inverse patterns for breast and prostate cancer may reflect more complex effects of screening-system maturity, public familiarity and offline information channels. These ecological associations should therefore be interpreted as exploratory, with additional analyses (considering differences in disease burden, screening practices and information environments as potential contributors) needed to confirm these mechanisms.

The decreasing search intensity trends for the studied cancer types were also evident during the COVID-19 pandemic, when search intensity decreased for all examined cancer types, although the magnitude of the decline varied across cancer types, suggesting a substantial reorientation of public attention. Similar declines in searches related to cancer and screening examinations during the early pandemic phase have been reported in several international studies utilizing GT data [36,38]. The reductions observed in our study may reflect the predominance of COVID-19-related information, shifts in media content, temporary limitations in screening and diagnostic services, and changes in healthcare utilization. This interpretation is supported by Hungarian healthcare delivery data from the NHIF, showing that organized breast and colorectal cancer screening programmes were temporarily suspended during the initial pandemic period [14,15], and that mammography coverage declined from 48.1 to 51.5% in 2012–2019 to 31.8% in the 2020–2021 screening cycle [14]. Thus, the decline in online cancer-related search interest occurred in parallel with measurable disruptions in cancer screening activity. However, the decrease in RSV cannot be interpreted directly as a change in cancer incidence, the number of diagnoses or screening participation, but rather as an indication of a pandemic-related shift in online public attention.

Analysis of search patterns around awareness campaigns showed that the “World Cancer Day” global campaign was not associated with notable changes in search intensity in Hungary, whereas among cancer-specific campaigns, only the Colorectal Cancer Awareness Campaign was accompanied by a substantial increase in search intensity. This cautions against the assumption that campaigns, by themselves, necessarily lead to increased online public interest. This contrast between institutional awareness campaigns and media events involving public figures is consistent with broader evidence that media exposure can shape preventive health attitudes, intentions and public responses, although these effects depend strongly on message credibility, audience characteristics and communication context [61]. Conversely, a lack of increased search activity does not necessarily indicate campaign ineffectiveness, as benefits may occur without additional Google^TM^ searches. Although direct causation between educational campaigns and health-related Internet search intensity is still poorly understood, previous studies have reported that campaigns with strong media support—particularly Breast Cancer Awareness Month—may trigger recurrent search peaks, although the magnitude and persistence of these effects have varied substantially between countries [35,39]. The observed campaign-associated changes are likely influenced not only by the topic, but also by visibility, timing, communication channels, concurrent media content and the underlying cultural context and social norms. This is especially relevant for campaigns held in November, when multiple cancer-related initiatives run in parallel, making it difficult to fully disentangle search changes associated with individual awareness periods. In our data, several pronounced peaks in search activity were not linked to organised campaigns, but rather to media coverage of cancer diagnoses and deaths of well-known public figures. For example, searches related to pancreatic cancer showed a marked increase in 2020 that coincided with the illness and death of a widely known individual. Similar phenomena have been documented in other infodemiological studies: public announcements of cancer diagnoses or deaths among celebrities may also produce short-term, sometimes substantial increases in online search intensity related to the corresponding cancer type, its screening, prognosis and treatment options [7,62,63]. These peaks illustrate how media coverage of highly visible public figures may temporarily override underlying long-term trends in cancer-related search activity, generating short-lived increases in information seeking that are distinct from the effects of planned awareness campaigns. Still, symptom-oriented information seeking may also be relevant to early-diagnosis goals, as better symptom knowledge and recognition of symptoms as potentially cancer-related have been associated with shorter help-seeking intervals [64]. The predominance of symptom-related queries should therefore not be interpreted solely as a gap in prevention-oriented information seeking. Future research should examine whether such searches prompt timely healthcare seeking and whether this translates into earlier cancer diagnosis. While the majority of published studies have highlighted this phenomenon in relation to cancers, demonstrable peaks in search intensity associated with other non-communicable diseases and rare conditions have also been described, following high-profile events or announcements by celebrities [65,66]. Overall, celebrity-related peaks in search activity demonstrate that the public responds rapidly to emotionally salient, personal cancer stories. Beyond search activity, a study of breast cancer communication found an indirect association between media use and screening intentions, mediated by identification with celebrities, attitudes and perceived social norms [67]. From a public health perspective, these events create brief windows of opportunity to disseminate accurate information and screening messages, but spontaneous media attention alone is unlikely to produce sustained behavioural change, unless it is coupled with well-planned, evidence-based communication campaigns.

The analysis of related queries revealed a clear dominance of symptom-oriented online information seeking within the topic of cancers. This may indicate that online information needs are particularly pronounced in relation to symptoms and the possibility of disease, whereas proactive information seeking about screening attendance, prevention and early detection is much less prominent. It is particularly noteworthy that for breast and colorectal cancer—both of which are covered by organised screening programmes—screening-related search terms did not consistently rank among the leading queries. This pattern aligns only partially with public health goals that regard prevention, early diagnosis and equitable access to organised screening as key pillars of cancer control [68,69,70]. Moreover, a substantial number of cancer-related queries focused on the perceived role of psychological factors in cancer development; this interest in presumed psychological causes is particularly noteworthy, as established cancer risk factors, such as tobacco use, obesity, unhealthy dietary patterns or chemical exposures—which are supported by robust epidemiological evidence [12]—were not present among the leading related queries. This pattern may indicate a cancer-related health literacy gap: while some users searched for non-evidence-based explanations, established modifiable risk factors had limited visibility among the leading queries. Therefore, digital cancer communication should more explicitly address evidence-based risk determinants and common misconceptions of the public. At the same time, the absence of established risk factors among the leading related queries does not necessarily indicate limited awareness of these factors, as search patterns may also reflect users’ existing knowledge. Similarly, searches concerning presumed psychological causes cannot be interpreted as evidence that users endorse such explanations. Our findings therefore do not simply indicate low interest in screening, but also suggest a communication gap in which population information needs and the emphasis of public health messages do not fully overlap. In this context, GT should be considered not as a direct measure of population knowledge, but as a complementary tool for identifying digital information demands and potential gaps in cancer-related health communication.

### 4.1. Strengths and Limitations of Our Study

A key strength of our study is the ten-year observation period, the parallel analysis of five cancer types of major public health relevance, and the placement of Hungarian results within a global context, complemented by an analysis of the possible changes during the COVID-19 period, thematic awareness campaigns and the content of related searches to ascertain search intent. The analysis of long-term trends based not only on RSV_raw_ values but also on adjusted RSV_sens_ values corrected for changes in the ratio of Internet users allowed us to assess whether observed search trends persisted after accounting for the increasing use of the Internet over time. The detailed documentation of query and data-processing procedures further improves the transparency and reproducibility of the present research, based on GT data analytics [27,29,36].

Nonetheless, our findings should be interpreted in light of several limitations. GT provides relative, normalized search values rather than absolute search counts, and does not include information on the demographic characteristics, health status or motivations of individuals performing the searches. The correction (in the form of RSV_sens_ values) introduced for the temporal changes in the ratio of Internet users should also be interpreted cautiously: due to the fact that GT provides normalized rather than absolute search volumes, adjusting RSV values by the correction factor introduced (to account for the change in Internet users compared to 2013) represents an investigator-derived adjustment rather than a formally validated procedure of GT. Therefore, RSV_sens_ estimates should be considered a sensitivity analysis alongside the uncorrected RSV_raw_ results, rather than as direct estimates of population-level search rates. A search query therefore cannot be directly equated with knowledge, awareness, screening participation or use of healthcare services. Search intensity may also be strongly influenced by media coverage, public events and the illnesses of well-known individuals, which limits the suitability of GT data for estimating or predicting the true epidemiological situation [30,36].

In our global analyses, an additional limitation is that Google^TM^ services may have been partially or completely blocked or heavily restricted in some countries (such as mainland China) during the study period, which may distort search data for these regions, and affect the global patterns observed. Moreover, differences in Google^TM^ availability, market share and search engine preferences across countries may also influence both country rankings and correlations between RSV values and HDI. Therefore, these country-level comparisons, together with RSV–HDI correlations, should be interpreted as exploratory contextual indicators, rather than precise measures of cross-national information demand. Additionally, Hungarian analyses were based on exact local-language search terms, whereas global analyses used multilingual topic-based queries. Because “search terms” do not capture synonyms, spelling variants or related derived forms of the relevant words, Hungarian search interest may have been underestimated relative to topic-based global estimates. The same caution applies to absolute RSV values, country rankings and international comparisons based on the “search term”–topic comparisons. Comparisons focusing on the COVID-19 period and awareness campaign periods are observational in nature, and causal relationships cannot be inferred from the observed changes in search intensity. Interpretation of campaign-associated changes may also be complicated by concurrent awareness activities, media coverage and other cancer-related events, which cannot be fully disentangled using GT data alone.

Finally, our analysis relied exclusively on Google^TM^ Web Search data. This may introduce platform selection bias, as older adults with a higher cancer burden may be underrepresented in online search data, while younger users may increasingly seek health information through social media or emerging artificial intelligence-based tools; in Hungary, 23.0% of respondents aged 15–29 years reported social media sites as starting points for health-related enquiries [55,56]. Therefore, our findings primarily reflect Google^TM^-based search behaviour and do not fully capture the broader digital health information environment [24,32,33]. In addition, GT relies on sampled search data, and repeated retrievals of identical queries may therefore yield slightly different RSV values, introducing a degree of sampling variability [27,36].

### 4.2. Public Health Implications

Our findings indicate that cancer-related online information seeking in Hungary is predominantly reactive and symptom-oriented, while searches related to screening and early detection are much less frequent. This is particularly concerning for breast and colorectal cancer, where organised screening programmes are readily available, yet screening-related search terms did not consistently rank among the leading queries. Current cancer control strategies of the WHO and the EU emphasise the reduction of major modifiable risk factors, early diagnosis, and equitable access to organised screening as key tools for reducing mortality [68,69,70,71]. In this context, GT analyses may serve as a useful complementary tool to identify topics that attract low spontaneous public interest, and areas where more targeted, accessible and intensive digital communication may be needed.

The heterogeneous and often limited impact of awareness campaigns further highlights that the success of public health communication strategies cannot be evaluated solely by the existence of a campaign, or the volume of associated media coverage. Evidence from populations with intellectual disabilities, for example, suggests that appropriately designed educational interventions may improve cancer-related awareness and screening-related outcomes [72]. Future campaigns should ideally be assessed using predefined digital and service-related indicators, such as search trends, website visits, screening appointment bookings and participation data, to allow their assessment in relation to a benchmark or a reference value. Regular monitoring of search queries could also aid public health authorities to respond more rapidly to infodemics involving inaccurate or non-evidence-based health information, and the information needs associated with them. In this role, infodemiological data should be considered not as a replacement for traditional epidemiological or health service data, but rather a complementary resource to support the planning and evaluation of cancer communication strategies. These patterns and their implications for major cancer types in Hungary are summarised in Table 8.

## 5. Conclusions

Health-related information-seeking from various online sources and the “digitally informed” patient are increasingly becoming the norm, with infodemiological studies allowing public health professionals to respond more dynamically to population concerns and behaviours shaped by the internet. Based on GT data from 2013 to 2022, online interest corresponding to the major cancer types studied in Hungary showed a long-term decline, with an additional decrease during the COVID-19 pandemic period. General cancer awareness campaigns were not associated with measurable changes in search intensity, whereas among the cancer-specific campaigns analysed, only the colorectal cancer-related campaigns were associated with a notable increase in search intensity. Related queries were predominantly symptom-focused, while searches about screening, prevention and early detection were detected much less frequently. Our findings suggest that public online information-seeking, as captured by exact Hungarian search terms in Google^TM^, only partially aligns with local epidemiological realities and population-level goals for cancer prevention and early detection. However, international comparisons should be interpreted cautiously, as Hungarian analyses were based on search terms, whereas global comparisons used multilingual topic-based queries. Although GT cannot be used as a direct measure of cancer incidence, mortality, population knowledge, awareness, screening uptake or health behaviour, it may provide complementary insights into online public attention and possible indicators of information needs or communication gaps. Future studies should link online search data with screening participation, healthcare utilisation and survey-based measures of knowledge and attitudes to better understand how changes in digital interest relate to actual health behaviours. From an operational perspective, continuous infodemiological monitoring could be integrated into national public health surveillance dashboards and interpreted concurrently with National Cancer Registry, screening participation and healthcare utilisation data. Such an infoveillance approach may help public health authorities identify possible emerging information gaps earlier and evaluate the online visibility of cancer prevention and health promotion campaigns more dynamically.

## Figures and Tables

**Table 1:** Global comparison and Hungary’s position in country rankings, for cancer-related information-seeking in Google Trends^TM.^

Lung Cancer Position/Number of Countries	Breast Cancer Position/Number of Countries	Prostate Cancer Position/Number of Countries	Colorectal Cancer Position/Number of Countries	Pancreatic Cancer Position/Number of Countries
1/50 China (RSV: 100)	1/74 Japan (RSV: 100)	1/72 Panama (RSV: 100)	1/67 Hong Kong (RSV: 100)	1/60 Republic of Korea (RSV: 100)
2/50 Taiwan (RSV: 93)	2/74 Ghana (RSV: 81)	2/72 Ghana (RSV: 87)	2/67 United Kingdom (RSV: 99)	2/60 Japan (RSV: 92)
3/50 Hong Kong (RSV: 89)	3/74 Lebanon (RSV: 80)	3/72 Peru (RSV: 84)	3/67 Taiwan (RSV: 97)	3/60 United States (RSV: 71)
…	…	…	…	…
29/50 Finland (RSV: 48)	71/74 Czechia (RSV: 18)	65/72 Greece (RSV: 16)	62/67 Brazil (RSV: 19)	28/60 Israel (RSV: 36)
**30/50 Hungary** **(RSV: 45)**	**72/74 Hungary** **(RSV: 18)**	**66/72 Hungary** **(RSV: 16)**	**63/67 Hungary** **(RSV: 19)**	**29/60 Hungary** **(RSV: 31)**
31/50 Romania (RSV:45)	73/74 Russia (RSV: 17)	67/72 India (RSV: 14)	64/67 Russia (RSV: 18)	30/60 Peru (RSV: 30)
…	…	…	…	…
50/50 Kazakhstan (RSV: 31)	74/74 Ukraine (RSV: 15)	72/72 Ukraine (RSV: 10)	67/67 Ukraine (RSV: 13)	60/60 Indonesia (RSV: 6)

Only countries with annual RSV ≥ 1 at least once during the study period for the respective cancer-related topic were included in the ranking.

**Table 2:** Correlation analysis (r_p_) between global online search intensity for cancers of public health importance (expressed as RSV) and country-level Human Development Index (HDI), 2013–2022.

	Lung Cancer	Breast Cancer	Prostate Cancer	Colorectal Cancer	Pancreatic Cancer
Analysis only including countries with annual RSV ≥ 1	*r* = 0.312 *p* = 0.216 *q* = 0.243	*r* = −0.032 *p* = 0.727 *q* = 0.727	*r* = −0.274 *p* = 0.019 *q* = 0.028	*r* = 0.405 *p* < 0.001 *q* < 0.001	*r* = 0.596 *p* < 0.001 *q* < 0.001
Analysis including all countries and territories	*r* = 0.518 *p* < 0.001 *q* < 0.001	*r* = −0.183 *p* = 0.043 *q* = 0.055	*r* = −0.573 *p* < 0.001 *q* < 0.001	*r* = 0.642 *p* < 0.001 *q* < 0.001	*r* = 0.669 *p* < 0.001 *q* < 0.001

*p*-values correspond to unadjusted, while *q*-values correspond to BH-adjusted results.

**Table 3:** Correlation analysis (r_p_) between online search intensity for cancers of public health importance in Hungary, 2013–2022 *.

	Breast Cancer	Lung Cancer	Colorectal Cancer	Prostate Cancer	Pancreatic Cancer
**Breast cancer**	X	*p* < 0.001 *q* < 0.001	*p* < 0.001 *q* < 0.001	*p* = 0.024 *q* = 0.038	*p* = 0.648 *q* = 0.720
**Lung cancer**	*r* = 0.339	X	*p* < 0.001 *q* < 0.001	*p* < 0.001 *q* < 0.001	*p* = 0.296 *q* = 0.422
**Colorectal cancer**	*r* = 0.365	*r* = 0.399	X	*p* = 0.082 *q* = 0.137	*p* = 0.697 *q* = 0.720
**Prostate cancer**	*r* = 0.207	*r* = 0.342	*r* = 0.162	X	*p* = 0.556 *q* = 0.695
**Pancreatic cancer**	*r* = −0.042	*r* = 0.119	*r* = 0.036	*r* = 0.054	X

* Correlation coefficients (r_p_) are presented below the diagonal and corresponding *p*-values (unadjusted) and *q*-values (BH-adjusted) above the diagonal.

**Table 4:** Long-term trends and seasonality in Google search intensity for selected cancer types in Hungary, 2013–2022.

Cancer Type	Linear Regression Slope (*β*)	Mann–Kendall S	Mann–Kendall *p*-Value	WWR *p*-Value	AAPC_2013–2022_ [95% CI]	AAPC *p*-Value	Seasonality Kruskal–Wallis *p*-Value
***RSV*** **_raw_**							
Lung cancer	−0.0078	−2960	<0.001	0.010	−4.14% [−7.24–−0.94]	0.017	0.296
Breast cancer	−0.0041	−1763	<0.001	0.0065	−2.27% [−5.36–0.92]	0.130	<0.001 (weeks 40–46)
Colorectal cancer	−0.0034	−1395	0.001	0.272	-6.59% [−16.12–−3.07]	0.002	0.806
Prostate cancer	−0.0022	−683	0.122	0.003	−1.56% [−2.92–0.12]	0.072	<0.001 (weeks 37–52)
Pancreatic cancer	−0.0017	−2007	<0.001	0.007	−6.97% [−10.87–−2.91]	0.004	0.374
***RSV_sens_***							
Lung cancer	−0.0086	−473	<0.001	0.009	−5.39% [−9.32–−1.29]	0.016	0.432
Breast cancer	−0.0074	−2569	<0.001	0.006	−4.06% [−7.69–−0.28]	0.038	<0.001 (weeks 40–46)
Colorectal cancer	−0.0062	−1639	<0.001	0.118	−3.56% [−6.28–−0.77]	0.018	0.902
Prostate cancer	−0.0102	−2385	0.022	0.001	-3.59% [−5.71–−1.44]	0.005	<0.001 (weeks 37–52)
Pancreatic cancer	−0.0022	−2519	<0.001	0.070	−5.23% [−10.53–−0.39]	0.049	0.501

RSV_raw_: raw/uncorrected relative search volume; RSV_sens_: investigator-derived RSV values used in the Internet penetration-adjusted sensitivity analysis; β: linear regression slope; S: Mann–Kendall test statistic, with negative and positive values indicating decreasing and increasing monotonic trends, respectively; WWR: Wald–Wolfowitz runs test; AAPC: average annual percent change; CI: confidence interval. Seasonality was assessed using the Kruskal–Wallis H test.

**Table 5:** Comparison of cancer-related Google search intensity before and during the COVID-19 period in Hungary, 2017–2019 vs. 2020–2022.

Cancer Type	Pre-COVID-19 (2017–2019) Mean RSV [95% CI]	COVID-19 Period (2020–2022) Mean RSV [95% CI]	Absolute Change (ΔRSV)	Relative Change (Δ%)	*p*-Value (*q*-Value)
**Lung cancer**	67.22 [65.47–68.98]	46.89 [45.13–48.64]	−20.33	−30.2%	<0.001 (<0.001)
**Breast cancer**	65.5 [63.48–67.52]	52.19 [50.18–54.21]	−13.31	−20.3%	<0.001 (<0.001)
**Prostate cancer**	61.64 [59.29–63.98]	50.81 [48.48–53.13]	−10.83	−17.6%	<0.001 (<0.001)
**Colorectal cancer**	53.69 [51.34–56.05]	48.25 [45.80–50.70]	−5.44	−10.1%	0.043 (0.053)
**Pancreatic cancer**	11.61 [9.76–13.46]	9.69 [7.85–11.53]	−1.92	−16.5%	0.501 (0.501)

RSV: relative search volume; CI: confidence interval; ΔRSV: absolute change in mean RSV between the COVID-19 and pre-COVID-19 periods; Δ%: relative percentage change in mean RSV. Values are presented as mean RSV with 95% CIs. *p*-values were obtained using paired-samples *t*-tests comparing paired observations from the pre-COVID-19 (2017–2019) and COVID-19 (2020–2022) periods; *q*-values were obtained following BH correction for pairwise analyses.

**Table 6:** Changes in online cancer-related search intensity associated with (A) World Cancer Day and (B) cancer-specific awareness campaigns in Hungary, 2013–2022.

Cancer Type	World Cancer Day Educational Campaign (A)		Absolute Change (ΔRSV)	Relative Change (Δ%)	*p*-Value (*q*-Value)
	Pre-Campaign Mean RSV [95% CI]	Campaign/Post Mean RSV [95% CI]			
Lung cancer	62.15 [56.63–67.77]	65.83	+3.68	+5.9%	0.321 (0.535)
		[61.32–70.33]			
Breast cancer	48.23 [40.93–55.52]	52.58	+4.35	+9.0%	0.336 (0.535)
		[47.13–58.02]			
Prostate cancer	49.33 [41.06–57.59]	47.28	−2.05	−4.20%	0.891 (0.933)
		[39.50–55.04]			
Colorectal cancer	29.48 [18.68–40.27]	28.55	−0.93	−3.2%	0.721 (0.801)
		[17.66–39.44]			
Pancreatic cancer	15.03 [6.30–23.75]	7.1	−7.93	−52.8%	0.096 (0.188)
		[0.30–13.90]			
**Cancer type**	**Cancer-specific awareness campaigns (B)**		**Absolute change** **(**Δ**RSV)**	**Relative change (**Δ**%)**	***p*** **-value** **(** ***q*** **-value)**
	**Pre-campaign** **mean RSV** **[95% CI]**	**Campaign/post** **mean RSV** **[95% CI]**			
Lung cancer	60.13 [54.20–66.05]	60.45 [53.61–67.29]	+0.32	+0.5%	0.933 (0.933)
Breast cancer	54.18 [47.13–61.23]	62.08 [54.33–69.82]	+7.9	+14.60%	0.113 (0.276)
Prostate cancer	47.28 [39.51–55.04]	48.34 [40.67–56.01]	+1.06	+2.20%	0.323 (0.535)
Colorectal cancer	26.58 [15.98–37.17]	44.35 [33.04–55.66]	+**17.77**	+**66.90%**	**0.013** (0.058)
Pancreatic cancer	12.23 [3.53–20.92]	19.18 [9.38–28.97]	+6.95	+56.8%	0.287 (0.535)

RSV: relative search volume; CI: confidence interval; ΔRSV: absolute change in mean RSV; Δ%: relative percentage change in mean RSV. For World Cancer Day (A), mean RSV values were compared between the week preceding World Cancer Day and the campaign week, in addition to the subsequent three-week latency period. For cancer-specific awareness campaigns (B), mean RSV values were compared between the calendar month immediately preceding each campaign and the respective awareness month. *p*-values were obtained using paired-sample *t*-tests comparing the corresponding predefined periods; *q*-values were obtained following BH correction for pairwise analyses.

**Table 7:** Presence of main search intent categories in cancer-related queries by cancer type in Hungary, 2013–2022.

Cancer Type	Symptoms	Treatment	Disease Prognosis/Stage	Screening/Early Detection	Dominant Intent
Lung cancer	✓	✓	✓	–	**Symptoms**
Breast cancer	✓	–	✓	✓	**Symptoms**
Colorectal cancer	✓	–	✓	✓	**Symptoms**
Prostate cancer	✓	✓	✓	✓	**Symptoms**
Pancreatic cancer	✓	–	✓	–	**Symptoms**

✓ indicates that at least one query corresponding to the respective search intent category was identified among the top related queries during the study period; “–” indicates that no such query was identified. Search queries were classified into predefined intent categories: symptoms, screening and early detection, treatment, disease prognosis, and other cancer-related information. Detailed annual term-level RSV data, including the original Hungarian search queries, are presented in Supplementary Material S5. RSV: relative search volume.

**Table 8:** Public health implications of cancer-related online information-seeking patterns in Hungary.

Cancer	Disease Burden Prognosis	Screening Status in Hungary	Early Detection/Screening Modality	Screening Search Visibility	Campaign Responsiveness	Main Finding
**Breast**	High	Organized nationwide screening since 2002 [59]	Mammography	Medium	No significant change	Symptoms dominate, despite availability of organised screening.
**Colorectal**	High	Organized programme introduced in 2018 [5]	Faecal immunochemical test followed by colonoscopy	Medium	Significant increase	Symptoms dominate despite availability of organised screening; significant campaign-associated increase observed (no longer significant, following correction for multiple testing).
**Lung**	Very high	No population-based organized screening programme; LDCT evaluated through HUNCHEST [71]	Low-dose CT in high-risk populations	Low	Not assessed *	Searches focus on symptoms rather than prevention.
**Prostate**	High	Opportunistic	PSA-based testing/clinical assessment	Medium	Not assessed *	Campaigns increase awareness, more than screening interest.
**Pancreatic**	High fatality	No organized population-based screening programme	No established population screening modality	Low	Not assessed *	Limited preventive information seeking.

*Note:* Screening search visibility is based on relative search volume categories, as defined in the Methods section. Campaign responsiveness indicates whether a statistically significant change in search intensity was observed during the cancer-specific awareness campaign periods analysed. World Cancer Day was analysed separately as a general cancer awareness campaign and was not associated with a statistically significant change in search intensity. *“Not assessed” indicates cancer types for which no cancer-specific awareness campaign was included in the campaign analysis. Abbreviations: CT: computer tomography; LDCT: low-dose computer tomography; PSA: prostate-specific antigen.

## Data Availability

Raw search intensity data from the Google Trends™ database are publicly available through Google Trends. https://trends.google.com/trends/ (accessed on 19 August 2026). Analysed data following data management, cleaning and processing are available from the corresponding author upon reasonable request.

## Supplementary Materials

The following supporting information can be downloaded at https://www.mdpi.com/article/10.3390/medsci14060681/s1; Supplementary Material S1: Ratio of Internet users and year-specific adjustment factors used for the Internet penetration-adjusted sensitivity analysis (RSV_sens_) in Hungary and worldwide, 2013–2022; Supplementary Material S2: STROBE checklist for observational studies; Supplementary Material S3: Worldwide country rankings of Google Trends^TM^ search interest for the five cancer-related topics, 2013–2022. Supplementary Material S4: Long-term global trends in Google^TM^ search intensity for selected cancer types, 2013–2022; Supplementary Material S5: Annual relative search volume (RSV) of cancer-related top queries by search intent category in Hungary, 2013–2022.

## References

[1] GBD 2023 Cancer Collaborators (2025). The global, regional, and national burden of cancer, 1990–2023, with forecasts to 2050: A systematic analysis for the Global Burden of Disease Study 2023. Lancet.

[2] Dou C., Sang Y., Huang L., Cao C. (2026). Burden of 33 malignant tumor groups in 5 socio-demographic index areas and 21 regions from 1990 to 2041. Sci. Prog..

[3] International Agency for Research on Cancer Global Cancer Observatory: Cancer Today. https://gco.iarc.fr/today/en.

[4] National Institute of Oncology National Cancer Registry and Biostatistics Center. https://onkol.hu/nemzeti-rakregiszter-es-biostatisztikai-kozpont/.

[5] OECD, European Commission (2025). EU Country Cancer Profile: Hungary 2025.

[6] Kiss Z., Bogos K., Tamási L., Ostoros G., Müller V., Bittner N., Sárosi V., Vastag A., Knollmajer K., Várnai M. (2022). Underlying reasons for post-mortem diagnosed lung cancer cases—A robust retrospective comparative study from Hungary (HULC study). Front. Oncol..

[7] Gianfredi V., Nucci D., Nardi M., Santangelo O.E., Provenzano S. (2023). Using Google Trends and Wikipedia to investigate the global public’s interest in the pancreatic cancer diagnosis of a celebrity. Int. J. Environ. Res. Public Health.

[8] MacDonald W.J., Purcell C., Pinho-Schwermann M., Stubbs N.M., Srinivasan P.R., El-Deiry W.S. (2025). Heterogeneity in Cancer. Cancers.

[9] Siegel R.L., Kratzer T.B., Wagle N.S., Sung H., Jemal A. (2026). Cancer statistics, 2026. CA Cancer J. Clin..

[10] Rozengurt E., Eibl G. (2026). Pancreatic cancer: Molecular pathogenesis and emerging therapeutic strategies. Signal Transduct. Target. Ther..

[11] Kenessey I., Nagy P., Polgár C. (2022). A rosszindulatú daganatok hazai epidemiológiai helyzete a XXI. század második évtizedében. Magy. Onkol..

[12] Parada M.L., Horn J., Cambron C. (2025). Examining the continuity of modifiable cancer-risk behaviors from youth into adulthood through prospective longitudinal studies: A scoping review. Soc. Sci. Med..

[13] World Health Organization Regional Office for Europe (2022). A Short Guide to Cancer Screening: Increase Effectiveness, Maximize Benefits and Minimize Harm.

[14] Laczó A., Bódis J., Bogner P., Molnár K., Vajda R., Pónusz-Kovács D., Elmer D., Kajos L.F., Csákvári T., Kívés Z. (2022). A szervezett lakossági mammográfiás emlőszűrés részvételi mutatói 2012–2021 között Magyarországon. Magy. Onkol..

[15] Kívés Z., Bódis J., Hunyady B., Pónusz-Kovács D., Elmer D., Kajos L.F., Csákvári T., Vajda R., Boncz I. (2022). A kolorektális szűrések részvételi mutatói Magyarországon 2008–2021 között. Magy. Onkol..

[16] Leonforte L., Mistretta A., Nicosia V., Micalizzi M.C., Londrigo D., Giambria M.M., Roscitano G., Veroux P., Veroux M. (2025). Epidemiological Overview of Colorectal Cancer in Kidney Transplant Recipients: A Systematic Review. Cancers.

[17] Elek P., Csanádi M., Fadgyas-Freyler P., Gervai N., Oross-Bécsi R., Szécsényi-Nagy B., Tatár M., Váradi B., Zemplényi A. (2022). Heterogeneous impact of the COVID-19 pandemic on lung, colorectal and breast cancer incidence in Hungary: Results from time series and panel data models. BMJ Open.

[18] Elek P., Fadgyas-Freyler P., Váradi B., Mayer B., Zemplényi A., Csanádi M. (2022). Effects of lower screening activity during the COVID-19 pandemic on breast cancer patient pathways: Evidence from the age cut-off of organized screening. Health Policy.

[19] Bdair I.A., Maribbay G.M.L., Bdair O.A., Maharmeh M., Alalyani M.M., Dator W.L.T., Tolentino A.D., Gerbolinga R.R., Darawad M.W. (2025). Awareness of cancer risk factors, protective factors, symptoms and barriers to seek medical advice among adult population. Asian Pac. J. Cancer Prev..

[20] Sękowski K., Jankowski M., Surma S., Olearczyk A., Zgliczyński W.S., Grudziąż-Sękowska J. (2026). Public awareness of cancer symptoms, risk factors, and prevention strategies among adults in Poland: A nationwide cross-sectional survey. Cancers.

[21] Ghanayem A., Elewa M., Abu Dawood H., Owais T.A., Ghanayem R., Almokdad H.K., Eltewacy N.K., EARG Collaborators (2026). Public awareness of colorectal cancer symptoms and risk factors, and exploring screening barriers across nine countries: A multi-national cross-sectional study. PLoS Glob. Public Health.

[22] Molnár R., Erdős C., Paulik E., Müller A., Nagymajtényi L. (2011). [A szűrővizsgálatokkal kapcsolatos információszerzés jellemzői a Dél-Alföldön] (article in Hungarian). Egészségfejlesztés/Hung. Health Prom. J..

[23] Fink H., Langselius O., Vignat J., Rumgay H., Rehm J., Martinez R.X., Santero M., Lopez-Perez L., Inoue M., Zeng H. (2026). Global and regional cancer burden attributable to modifiable risk factors to inform prevention. Nat. Med..

[24] Wang X., Shi J., Kong H. (2021). Online health information seeking: A review and meta-analysis. Health Commun..

[25] World Health Organization (2020). An Ad Hoc WHO Technical Consultation Managing the COVID-19 Infodemic: Call for Action, 7–8 April 2020.

[26] Eysenbach G. (2009). Infodemiology and infoveillance: Framework for an emerging set of public health informatics methods to analyze search, communication and publication behavior on the Internet. J. Med. Internet Res..

[27] Mavragani A., Ochoa G. (2019). Google Trends in infodemiology and infoveillance: Methodology framework. JMIR Public Health Surveill..

[28] StatCounter Global Stats. Search Engine Market Share Worldwide. https://gs.statcounter.com/search-engine-market-share.

[29] Nuti S.V., Wayda B., Ranasinghe I., Wang S., Dreyer R.P., Chen S.I., Murugiah K. (2014). The use of Google Trends in health care research: A systematic review. PLoS ONE.

[30] Cervellin G., Comelli I., Lippi G. (2017). Is Google Trends a reliable tool for digital epidemiology? Insights from different clinical settings. J. Epidemiol. Glob. Health.

[31] Hughes T., Harper A., Gupta S., Frazier A.L., van der Graaf W.T.A., Moreno F., Joseph A., Fidler-Benaoudia M.M. (2024). The current and future global burden of cancer among adolescents and young adults: A population-based study. Lancet Oncol..

[32] Jia X., Pang Y., Liu L.S. (2021). Online health information seeking behavior: A systematic review. Healthcare.

[33] Stifjell K., Sandanger T.M., Wien C. (2025). Exploring online health information-seeking behavior among young adults: Scoping review. J. Med. Internet Res..

[34] Phillips C.A., Barz Leahy A., Li Y., Schapira M.M., Bailey L.C., Merchant R.M. (2018). Relationship between state-level Google online search volume and cancer incidence in the United States: Retrospective study. J. Med. Internet Res..

[35] Vasconcellos-Silva P.R., Carvalho D.B.F., Trajano V., de La Rocque L.R., Sawada A.C.M.B., Juvanhol L.L. (2017). Using Google Trends data to study public interest in breast cancer screening in Brazil: Why not a Pink February?. JMIR Public Health Surveill..

[36] Kamiński M., Czarny J., Skrzypczak P., Sienicki K., Roszak M. (2023). The characteristics, uses, and biases of studies related to malignancies using Google Trends: Systematic review. J. Med. Internet Res..

[37] Eala M.A.B., Tantengco O.A.G. (2022). Global online interest in cervical cancer care in the time of COVID-19: An infodemiology study. Gynecol. Oncol. Rep..

[38] Tan S.Y., Tang M.S.S., Ong C.A.J., Tan V.K.M., Shannon N.B. (2023). Impact of COVID-19 on public interest in breast cancer screening and related symptoms: Google Trends analysis. JMIR Cancer.

[39] Ramadan M., Aboalola D., Aouabdi S., Alghamdi T., Alsolami M., Samkari A., Alsiary R. (2024). Influence of Breast Cancer Awareness Month on public interest of breast cancer in high-income countries between 2012 and 2022: Google Trends analysis. JMIR Cancer.

[40] Ádány R., Juhász A., Nagy C., Burkali B., Pikó P., McKee M., Oroszi B. (2024). Discrepancies between the spatial distribution of cancer incidence and mortality as an indicator of unmet needs in cancer prevention and/or treatment in Hungary. Cancers.

[41] Paguio J.A., Yao J.S., Reyes M.S.G.L., Lee G., Dee E.C. (2021). Bladder cancer and Google Trends: Associations between US search patterns and disease outcomes may show need for improved awareness strategies. J. Cancer Educ..

[42] Hungarian Central Statistical Office Internet Users as a Percentage of the Population Aged 16–74 Years. https://www.ksh.hu/stadat_files/ikt/hu/ikt0029.html.

[43] World Bank Individuals Using the Internet (% of Population). https://data.worldbank.org/indicator/IT.NET.USER.ZS.

[44] United Nations Development Programme Human Development Index (HDI). Human Development Data Center. https://hdr.undp.org/data-center/human-development-index#/indicies/HDI.

[45] Wang H.W., Chen D.R., Yu H.W., Chen Y.M. (2019). Evaluating the impact of health awareness events on Google search frequency. Prev. Med. Rep..

[46] McHugh M.L. (2012). Interrater reliability: The kappa statistic. Biochem. Med..

[47] von Elm E., Altman D.G., Egger M., Pocock S.J., Gøtzsche P.C., Vandenbroucke J.P., STROBE Initiative (2007). The Strengthening the Reporting of Observational Studies in Epidemiology (STROBE) statement: Guidelines for reporting observational studies. PLoS Med..

[48] World Summit Against Cancer for the New Millennium (2000). Charter of Paris Against Cancer.

[49] World Health Organization Breast Cancer Awareness Month 2025. https://www.who.int/news-room/events/detail/2025/10/01/default-calendar/breast-cancer-awareness-month-2025.

[50] Wang W., Xie Y., Liu X., Yan H., Chen Y., Wang L., Ning L. (2026). Facilitators and barriers to health information-seeking behavior in cancer patients: A meta-synthesis. Digit. Health.

[51] Jamiu M.O., Ozoeze U.N., Gbonjubola Y.T., Muhammad D.G. (2023). The pattern of online health information-seeking behavior before and during the COVID-19: An online cross-sectional survey among Nigerians. Sri Ramachandra J. Health Sci..

[52] Silburn A. (2025). Navigating online health information: Empowerment vs. misinformation. Front. Digit. Health.

[53] Van Riel N., Auwerx K., Debbaut P., Van Hees S., Schoenmakers B. (2017). The effect of Dr Google on doctor–patient encounters in primary care: A quantitative, observational, cross-sectional study. BJGP Open.

[54] Marcu A., Black G., Whitaker K.L. (2018). Variations in trust in Dr Google when experiencing potential breast cancer symptoms: Exploring motivations to seek health information online. Health Risk Soc..

[55] Eurostat Individuals Using the Internet for Seeking Health-Related Information. Eurostat Data Browser. https://ec.europa.eu/eurostat/databrowser/view/tin00101/default/table.

[56] (2024). Ifjúságkutató Intézet/MCC Egészségedre Program. A Megelőzés Mellett Voksolnak a Fiatalok. https://ifjusagkutatointezet.hu/kiadvany/a-megelozes-mellett-voksolnak-a-fiatalok.

[57] Eysenbach G. (2011). Infodemiology and infoveillance: Tracking online health information and cyberbehavior for public health. Am. J. Prev. Med..

[58] Hungarian Central Statistical Office Number and Rate of Deaths, Infant Mortality, Life Expectancy at Birth, Deaths by Main Causes of Death. STADAT Table 22.1.1.9. https://www.ksh.hu/stadat_files/nep/en/nep0009.html.

[59] Boncz I., Sebestyén A., Döbrőssy L., Péntek Z., Budai A., Kovács A., Dózsa C., Ember I. (2007). The organisation and results of first screening round of the Hungarian nationwide organised breast cancer screening programme. Ann. Oncol..

[60] Kirli Bolukbas M., Figen M., Kocak Uzel E. (2024). Cancer awareness across borders: A comparative study of internet search trends for breast and prostate cancer in Europe and Turkey. BMC Cancer.

[61] Leonforte F., Nicosia V., Comite P., Morlino G., Mistretta A. (2026). Media Exposure and Its Association with Vaccine Attitudes, Intentions, and Hesitancy: Systematic Review. J. Med. Internet Res..

[62] Kaleem T., Malouff T.D., Stross W.C., Waddle M.R., Miller D.H., Seymour A.L., Zaorsky N.G., Miller R.C., Trifiletti D.M., Vallow L. (2019). Google Search Trends in Oncology and the Impact of Celebrity Cancer Awareness. Cureus.

[63] Sajjadi N.B., Feldman K., Shepard S., Reddy A.K., Torgerson T., Hartwell M., Vassar M. (2021). Public interest and behavior change in the United States regarding colorectal cancer following the death of Chadwick Boseman: Infodemiology investigation of Internet search trends nationally and in at-risk areas. JMIR Infodemiology.

[64] Petrova D., Okan Y., Salamanca-Fernández E., Domínguez-López S., Sánchez M.J., Rodríguez-Barranco M. (2020). Psychological factors related to time to help-seeking for cancer symptoms: A meta-analysis across cancer sites. Health Psychol. Rev..

[65] Erickson K.L., Vaccarello A., Rohr B.R. (2023). The impact of celebrity influence on public interest in alopecia areata: A 10-year Google Trends analysis. Aesthet. Plast. Surg..

[66] Elsalti A., Darkhabani M., Alrifaai M.A., Mahroum N. (2023). Celebrities and medical awareness—The case of Celine Dion and stiff-person syndrome. Int. J. Environ. Res. Public Health.

[67] Cho J.S., Tian Y. (2024). Celebrity Identification and Reasoned Action: An Integrative Model of the Relationship Between Media Use and Breast Cancer Screening Intention. Health Commun..

[68] World Health Organization (2017). Guide to Cancer Early Diagnosis.

[69] European Commission (2021). Europe’s Beating Cancer Plan.

[70] Council of the European Union (2022). Council Recommendation of 9 December 2022 on strengthening prevention through early detection: A new EU approach on cancer screening replacing Council Recommendation 2003/878/EC (2022/C 473/01). Off. J. Eur. Union.

[71] Kerpel-Fronius A., Monostori Z., Kovacs G., Ostoros G., Horvath I., Solymosi D., Pipek O., Szatmari F., Kovacs A., Markoczy Z. (2022). Nationwide lung cancer screening with low-dose computed tomography: Implementation and first results of the HUNCHEST screening program. Eur. Radiol..

[72] Elmadani M., Klara S., Kiptulon E.K., Andrade D., Orsolya M. (2024). A systematic review of educational interventions to enhance cancer awareness and screening in individuals with intellectual disabilities: A mixed-methods approach. Res. Dev. Disabil..

