# Supplementary material for: Online Information-Seeking Behaviour for Major Cancers: A 10-Year Infodemiological Study to Inform Public Health and Health Promotion Priorities in Hungary
Source: Med Sci (Basel). 2026 Oct 17;14(6):681. doi: 10.3390/medsci14060681 (PMC13706818; doi:10.3390/medsci14060681)
Supplement: Supplementary file 1 [file medsci-14-00681-s001.zip › Supplementary Material S1.pdf]

Supplementary Material S1. Ratio of Internet users and year-specific sensitivity adjustment factors used for the Internet penetration-adjusted sensitivity analysis in Hungary and worldwide, 2013–2022 [1, 2]

Year	Hungary		Worldwide	
	**Internet users (%)**	**Sensitivity adjustment factor**	**Internet users (%)**	**Sensitivity adjustment factor**
**2013**	73.0	1.000	36.0	1.000
**2014**	76.0	1.041	38.0	1.056
**2015**	73.0	1.000	40.0	1.111
**2016**	79.0	1.082	43.0	1.194
**2017**	77.0	1.055	46.0	1.278
**2018**	76.0	1.041	49.0	1.361
**2019**	80.0	1.096	54.0	1.500
**2020**	85.0	1.164	60.0	1.667
**2021**	89.0	1.219	63.0	1.750
**2022**	90.0	1.233	63.0*	1.750

Note: Sensitivity adjustment factors were calculated using 2013 as the reference year by dividing the proportion of Internet users in each year by the corresponding proportion in 2013. Worldwide internet penetration data for 2022 were not available at the time of data collection; therefore, the 2021 value was used to calculate the correction factor for 2022.

1. Hungarian Central Statistical Office. Internet Users as a Percentage of the Population Aged 16–74 Years. Available online: https://www.ksh.hu/stadat_files/ikt/hu/ikt0029.html (accessed on 19 August 2026).
2. World Bank. Individuals Using the Internet (% of Population). Available online: https://data.worldbank.org/indicator/IT.NET.USER.ZS (accessed on 19 August 2026).
