# Supplementary material for: Online Information-Seeking Behaviour for Major Cancers: A 10-Year Infodemiological Study to Inform Public Health and Health Promotion Priorities in Hungary
Source: Med Sci (Basel). 2026 Oct 17;14(6):681. doi: 10.3390/medsci14060681 (PMC13706818; doi:10.3390/medsci14060681)
Supplement: Supplementary file 1 [file medsci-14-00681-s001.zip › Supplementary Material S2.pdf]

Supplementary Material S2. STROBE checklist for the present observational infodemiological study.

Item	Item No.	Recommendation / Reporting in the present study	Page No.
**Title and abstract**	**1(a)**	The study is explicitly described as a retrospective observational infodemiological study using Google Trends™ data.	1
	**1(b)**	The structured abstract summarizes the 2013–2022 Google Trends analysis of five cancers, statistical analyses, long-term trends, COVID-19-related changes, campaign effects, seasonality, and search-intent findings.	1-2
**Background/rationale**	**2**	The Introduction describes the global and Hungarian cancer burden, screening and prevention context, online health-information seeking, infodemiology, Google Trends methodology, and the need to examine whether online interest reflects cancer-related public health priorities.	2–5
**Objectives**	**3**	The study aimed to analyse online information-seeking related to lung, colorectal, breast, prostate, and pancreatic cancers in Hungary (2013–2022), compare findings with global trends, assess COVID-19 and awareness-campaign effects, examine major publicity events, and qualitatively assess search intent.	5
**Study design**	**4**	Retrospective, observational infodemiological study using Google Trends data, covering 1 January 2013 to 31 December 2022.	5
**Setting**	**5**	Hungary was the primary setting; worldwide Google Trends data were used for international contextual comparisons. The observation period was 2013–2022; raw GT data were collected between 19 February and 3 March 2024.	5
**Participants**	**6(a)**	No individual participants were enrolled. The study used anonymized, aggregated population-level Google Trends data.	—
	**6(b)**	Not applicable: no individual participants, matched cohorts, cases, or controls were used.	—
**Variables**	**7**	Main quantitative variables included RSV*raw*, internet penetration-corrected RSV (RSV*corr*), reference-adjusted RSV (RSV*ref*), HDI, calendar time, predefined COVID-19 periods, campaign periods, and media-event windows. Search-intent categories were also predefined.	6
**Data sources/measurement**	**8***	Google Trends™ supplied search-intensity data. Hungarian analyses used predefined search terms; global analyses used GT Topics. Internet-penetration data came from the Hungarian Central Statistical Office and World Bank, and HDI values from UNDP. Year-specific correction factors are documented in Supplementary Table S1.	6-7
**Bias**	**9**	Standardized Google Trends™ settings and predefined query procedures were used. Temporal changes in internet access were addressed using year-specific internet-penetration correction factors (RSV*corr*). Remaining platform, media-event, country-access, and search-term/topic limitations are discussed explicitly.	—
**Study size**	**10**	No participant sample size was calculated. Analyses used all available GT observations within the predefined 2013–2022 study period and countries/territories meeting the analysis-specific inclusion criteria.	—
**Quantitative variables**	**11**	RSV values were analysed as RSV*raw* and RSV*corr*; RSV*ref* was used for descriptive cross-cancer comparison. Absolute and relative changes were calculated for predefined comparison periods. Country inclusion thresholds, correlation-strength categories, COVID-19 periods, campaign windows, and event windows were predefined.	7

**Statistical methods**	**12(a)**	Analyses included descriptive statistics, linear regression, Mann–Kendall tests, Wald–Wolfowitz runs tests, Pearson correlations, paired-samples t-tests, one-way ANOVA with Tukey post hoc tests, Kruskal–Wallis tests, and Joinpoint regression with AAPCs and 95% CIs. Statistical significance was set at $p<0.05$.	8-9
	**12(b)**	No formal subgroup or interaction analyses were performed. Cancer types and predefined temporal/event comparisons were analysed separately.	—
	**12(c)**	No conventional missing-data imputation was performed. GT values of 0 indicated insufficient search volume for reporting. Countries/territories and related-query outputs were handled according to predefined analysis-specific availability and inclusion criteria.	—
	**12(d)**	Not applicable: no participant follow-up, matching, or survey sampling was undertaken.	—
	**12(e)**	No formal sensitivity analysis was performed. However, long-term trends were evaluated in parallel using RSV*raw* and internet penetration-corrected RSV*corr*, allowing assessment of whether trends persisted after correction for changing internet use.	—
**Participants**	**13(a)***	No individuals were enrolled. Figure 1 summarizes the study workflow, including query selection, data extraction, RSV processing/correction, global ranking, temporal analyses, and search-intent classification.	9
	**13(b)**	Not applicable: no individual participants were recruited.	—
	**13(c)**	Figure 1 presents the analytical workflow for data selection, extraction, processing, correction, and analysis.	9
**Descriptive data**	**14(a)***	Not applicable because no individual-level participant data were available in Google Trends.	—
	**14(b)**	Not applicable: no participant-level variables were analysed.	—
	**14(c)**	Not applicable: this was not a cohort study and there was no individual follow-up.	—
**Outcome data**	**15***	Results report relative search intensity, country rankings, HDI correlations, temporal trends, seasonality, COVID-19 changes, campaign responses, media-event changes, and search-intent patterns for the five cancer types.	9-19
**Main results**	**16(a)**	Results include RSV*raw*/RSV*corr* trend estimates, mean RSV with 95% CIs, absolute and relative changes, β coefficients, Mann–Kendall statistics and *p*-values, AAPCs with 95% CIs, correlation coefficients, and COVID-19/campaign comparison *p*-values.	8-19
	**16(b)**	Correlation strength was categorized as $\|r\|<0.3$ weak; $0.3\leq\|r\|<0.5$ moderate; $0.5\leq\|r\|<0.8$ strong; and $\|r\|\geq0.8$ very strong. Predefined temporal windows and country inclusion thresholds are also reported.	9
	**16(c)**	Not applicable: the study did not estimate relative risks or individual-level disease risks.	—
**Other analyses**	**17**	Additional analyses included HDI correlations, inter-cancer correlations, seasonality, COVID-19 comparisons, World Cancer Day and cancer-specific campaign analyses, high-profile media events, and qualitative search-intent classification.	11-12
**Key results**	**18**	Four principal findings are summarized: long-term decline in cancer-related search intensity; further decline during COVID-19; limited campaign responsiveness except for colorectal cancer; and dominance of symptom-focused over screening/prevention searches.	—

**Limitations**	**19**	Limitations include normalized rather than absolute GT values; lack of demographic, clinical, and motivational information; influence of media events; Google restrictions in some countries; differences between search terms and Topics; observational COVID-19/campaign comparisons; and omission of non-Google platforms.	23-24
**Interpretation**	**20**	Findings are interpreted as indicators of public online attention and information demand rather than direct measures of cancer incidence, mortality, knowledge, screening participation, or health behaviour. Results are compared with previous infodemiological studies and Hungarian cancer-control priorities.	24-25
**Generalisability**	**21**	Findings primarily reflect Google-based search behaviour and should not be considered representative of all online health-information seeking in Hungary. Use of other digital platforms and restricted Google access in some countries further limit generalisability of the national and global findings.	23-25
**Funding**	**22**	The research was supported by the "Serbian Scientific Cooperation Program with the Diaspora" (*Information-seeking behavior of the Serbian and Hungarian population related to cancers: comparative digital epidemiological research to determine the focal points of health promotion strategies*: INFO-EPICC; ID: 320) of the Science Fund of the Republic of Serbia.	26

*Give information separately for cases and controls in case-control studies and, if applicable, for exposed and unexposed groups in cohort and cross-sectional studies.

Note: An Explanation and Elaboration article discusses each checklist item and gives methodological background and published examples of transparent reporting. The STROBE checklist is best used in conjunction with this article (freely available on the Web sites of PLoS Medicine at http://www.plosmedicine.org/, Annals of Internal Medicine at http://www.annals.org/, and Epidemiology at http://www.epidem.com/). Information on the STROBE Initiative is available at www.strobe-statement.org.
