# Supplementary material for: Online Information-Seeking Behaviour for Major Cancers: A 10-Year Infodemiological Study to Inform Public Health and Health Promotion Priorities in Hungary
Source: Med Sci (Basel). 2026 Oct 17;14(6):681. doi: 10.3390/medsci14060681 (PMC13706818; doi:10.3390/medsci14060681)
Supplement: Supplementary file 1 [file medsci-14-00681-s001.zip › Supplementary Material S3.pdf]

Supplementary Table S3. Worldwide country rankings of Google Trends™ (GT) search interest for the five cancer-related topics, 2013–2022.

Rank	Country	RSV
**Lung cancer**		
1	China	100
2	Taiwan	93
3	Hong Kong	89
4	Japan	86
5	South Korea	76
6	United Kingdom	71
7	United States	68
8	Lebanon	67
9	Ireland	66
10	Canada	65
11	Vietnam	63
12	Germany	62
13	Philippines	62
14	Türkiye	60
15	Netherlands	60
16	Australia	58
17	Bosnia & Herzegovina	57
18	Singapore	57
19	Norway	56
20	Austria	55
21	Serbia	53
22	Croatia	52
23	Poland	50
24	Denmark	50
25	Switzerland	49
26	New Zealand	49
27	Spain	48
28	France	48
29	Finland	48
**30**	**Hungary**	**45**
31	Romania	45
32	Sweden	45
33	Tunisia	44
34	Belgium	44
35	South Africa	44
36	Bulgaria	42
37	Jordan	41
38	Greece	41
39	Iran	40
40	Thailand	38
41	Malaysia	37
42	Portugal	37
43	Indonesia	36
44	Italy	34
45	Algeria	34
46	Israel	34
47	Chile	33
48	Russia	33
49	Peru	33
50	Kazakhstan	31

Rank	Country	RSV
	**Breast cancer**	
1	Japan	100
2	Ghana	81
3	Lebanon	80
4	United States	71
5	Paraguay	68
6	United Kingdom	66
7	Taiwan	66
8	Ireland	65
9	Puerto Rico	64
10	Australia	63
11	Jordan	62
12	New Zealand	62
13	Panama	62
14	Mexico	61
15	Indonesia	59
16	Algeria	59
17	Nigeria	56
18	Canada	56
19	Hong Kong	56
20	Saudi Arabia	54
21	Singapore	54
22	Philippines	53
23	United Arab Emirates	53
24	Venezuela	53
25	Tunisia	51
26	Costa Rica	51
27	Iran	50
28	Egypt	48
29	Kenya	48
30	Dominican Republic	48
31	South Africa	46
32	Bolivia	46
33	Malaysia	46
34	China	44
35	Spain	42
36	Netherlands	42
37	Sweden	42
38	Chile	41
39	Finland	41
40	South Korea	40
41	Germany	40
42	Portugal	39
43	Norway	39
44	Switzerland	38
45	Pakistan	38
46	Israel	38
47	Brazil	37
48	Ecuador	37
49	Peru	37
50	Argentina	36
51	Guatemala	36
52	Austria	35
53	Iraq	34

Rank	Country	RSV
54	Colombia	34
55	Denmark	32
56	Poland	31
57	Morocco	31
58	Croatia	31
59	Belgium	31
60	France	30
61	Serbia	29
62	Thailand	29
63	Bulgaria	29
64	Greece	27
65	Vietnam	26
66	Türkiye	26
67	Romania	25
68	Italy	25
69	Bangladesh	24
70	India	22
71	Czechia	21
**72**	**Hungary**	**18**
73	Russia	17
74	Ukraine	15
**Prostate cancer**		
1	Panama	100
2	Ghana	87
3	Peru	84
4	Puerto Rico	84
5	Bolivia	82
6	Guatemala	75
7	El Salvador	74
8	Honduras	72
9	Venezuela	71
10	Costa Rica	69
11	Paraguay	68
12	Mexico	68
13	Ecuador	67
14	United Kingdom	66
15	Colombia	65
16	Chile	65
17	Kenya	62
18	Dominican Republic	61
19	Ireland	57
20	France	55
21	Algeria	55
22	Nigeria	55
23	Australia	54
24	United States	54
25	South Africa	53
26	Sweden	51
27	New Zealand	50
28	Canada	50
29	Norway	46
30	Uruguay	46
31	Tunisia	45
32	Spain	44

Rank	Country	RSV
33	Morocco	42
34	Argentina	41
35	Denmark	40
36	Switzerland	39
37	Belgium	39
38	Singapore	38
39	Philippines	38
40	Finland	37
41	Brazil	36
42	Netherlands	34
43	Germany	33
44	Portugal	33
45	Türkiye	32
46	Hong Kong	31
47	Japan	31
48	Taiwan	30
49	Austria	29
50	Indonesia	29
51	Italy	28
52	Poland	26
53	Malaysia	26
54	Croatia	24
55	Israel	24
56	Iran	24
57	United Arab Emirates	22
58	Slovakia	21
59	South Korea	20
60	Czechia	20
61	Serbia	20
62	Romania	18
63	Bulgaria	17
64	Pakistan	16
65	Greece	16
**66**	**Hungary**	**16**
67	India	14
68	Egypt	13
69	Saudi Arabia	12
70	Thailand	12
71	Russia	10
72	Ukraine	10
**Colorectal cancer**		
1	Hong Kong	100
2	United Kingdom	99
3	Taiwan	97
4	Japan	91
5	Australia	86
6	Ireland	78
7	New Zealand	78
8	China	74
9	Philippines	72
10	Singapore	72
11	South Korea	70
12	Paraguay	66
13	United States	66

Rank	Country	RSV
14	Canada	63
15	Spain	56
16	Norway	55
17	Jordan	52
18	Iran	51
19	Netherlands	51
20	Costa Rica	51
21	Romania	49
22	Croatia	49
23	Germany	48
24	Malaysia	48
25	Switzerland	47
26	Algeria	45
27	Peru	45
28	Argentina	44
29	Chile	44
30	Belgium	43
31	South Africa	43
32	Türkiye	42
33	Bulgaria	42
34	Mexico	41
35	Austria	40
36	Poland	40
37	Sweden	40
38	Dominican Republic	38
39	France	38
40	United Arab Emirates	36
41	Ecuador	36
42	Kenya	36
43	Saudi Arabia	35
44	Colombia	35
45	Egypt	34
46	Italy	33
47	Serbia	33
48	Nigeria	32
49	Bolivia	32
50	Venezuela	31
51	Israel	29
52	Denmark	28
53	Morocco	28
54	Portugal	27
55	Finland	26
56	Vietnam	25
57	Iraq	25
58	Bangladesh	25
59	Greece	23
60	Pakistan	20
61	Thailand	20
62	Brazil	19
**63**	**Hungary**	**19**
64	Russia	18
65	Indonesia	18
66	India	16
67	Ukraine	13

Rank	Country	RSV
**Pancreatic cancer**		
1	South Korea	100
2	Japan	92
3	United States	71
4	Taiwan	70
5	United Kingdom	69
6	Canada	61
7	Ireland	60
8	Finland	59
9	Australia	58
10	Italy	56
11	Switzerland	55
12	Germany	55
13	France	52
14	Austria	52
15	Poland	52
16	New Zealand	51
17	Hong Kong	50
18	China	50
19	Spain	44
20	Belgium	43
21	Singapore	43
22	Slovakia	41
23	Norway	40
24	Portugal	39
25	Türkiye	39
26	Netherlands	39
27	Sweden	39
28	Israel	36
**29**	**Hungary**	**31**
30	Peru	30
31	Chile	30
32	South Africa	30
33	Philippines	30
34	Czechia	29
35	Serbia	28
36	Iran	27
37	Ecuador	27
38	Argentina	27
39	Croatia	26
40	Mexico	25
41	Romania	25
42	Bulgaria	25
43	Greece	24
44	Malaysia	23
45	Algeria	22
46	Denmark	21
47	Brazil	21
48	Vietnam	21
49	Colombia	20
50	Venezuela	17
51	United Arab Emirates	17
52	Morocco	16
53	Russia	16

Rank	Country	RSV
54	Egypt	15
55	Ukraine	13
56	Saudi Arabia	12
57	Pakistan	11
58	India	11
59	Thailand	9
60	Indonesia	6

Note: Only countries with RSV ≥ 1 in at least one year during the study period for the respective cancer-related topic were included in the ranking. RSV, relative search volume. Worldwide data were retrieved using the Google Trends™ (GT) "Topic" function.
