# Supplementary material for: Online Information-Seeking Behaviour for Major Cancers: A 10-Year Infodemiological Study to Inform Public Health and Health Promotion Priorities in Hungary
Source: Med Sci (Basel). 2026 Oct 17;14(6):681. doi: 10.3390/medsci14060681 (PMC13706818; doi:10.3390/medsci14060681)
Supplement: Supplementary file 1 [file medsci-14-00681-s001.zip › Supplementary Material S4.pdf]

Supplementary Material S4. Long-term global trends in Google™ search intensity for selected cancer types, 2013–2022

Cancer type	Trend slope (β)	Mann–Kendall S, *p*-value	WWR *p*-value
Uncorrected RSV (RSV_{raw})			
**Lung cancer**	-0.0094	-530, 0.229	0.078
**Breast cancer**	-0.0006	-120, 0.786	0.776
**Colorectal cancer**	0.00029	+1353, <0.001	<0.001
**Prostate cancer**	0.0005	+33, 0.942	<0.001
**Pancreatic cancer**	0.0038	+3472, <0.001	<0.001
Internet penetration-adjusted sensitivity analysis for RSV (RSV_{sens})			
**Lung cancer**	-0.0102	-5326, <0.001	0.073
**Breast cancer**	-0.007	-4361, <0.001	0.733
**Colorectal cancer**	-0.0046	-1601, <0.001	<0.001
**Prostate cancer**	-0.0049	-3939, <0.001	<0.001
**Pancreatic cancer**	-0.0017	-4074, <0.001	<0.001

RSV: relative search volume; RSV_{raw}: raw (uncorrected) relative search volume; RSV_{sens}: investigator-derived RSV values used in the Internet penetration-adjusted sensitivity analysis β: linear regression slope, S: Mann–Kendall test statistic, with negative and positive values indicating decreasing and increasing monotonic trends, respectively; WWR: Wald–Wolfowitz runs test. RSV_{sens} values were adjusted for changes in the proportion of Internet users over the study period. Mann–Kendall *p*-values indicate the statistical significance of monotonic temporal trends.
