# Supplementary material for: Online Information-Seeking Behaviour for Major Cancers: A 10-Year Infodemiological Study to Inform Public Health and Health Promotion Priorities in Hungary
Source: Med Sci (Basel). 2026 Oct 17;14(6):681. doi: 10.3390/medsci14060681 (PMC13706818; doi:10.3390/medsci14060681)
Supplement: Supplementary file 1 [file medsci-14-00681-s001.zip › Supplementary Material S5.pdf]

Supplementary Material S5. Annual relative search volume of cancer-related top queries by search intent category in Hungary, 2013–2022

Search query	Intent category	2013	2014	2015	2016	2017	2018	2019	2020	2021	2022
Lung cancer											
"tüdőrák tünetei"	Symptoms	100	100	100	100	100	100	100	100	100	100
"tüdőrák jelei"	Symptoms	31	32	34	43	44	27	48	35	19	19
"tüdőrák tünetek"	Symptoms	–	–	12	15	–	5	9	5	3	–
"kissejtes tüdőrák"	Other cancer-related information	18	12	13	21	18	11	25	19	24	20
"tüdőrák kezelése"	Treatment	16	12	9	13	12	10	11	7	10	9
"tüdőgyulladás"	Other cancer-related information	12	9	10	11	9	7	–	10	–	–
"tüdőrák gyógyítása"	Treatment	11	7	7	9	8	–	8	–	10	5
"tüdőrák stádiumai"	Disease prognosis	10	7	10	4	5	4	5	5	8	8
"tüdőrák lefolyása"	Disease prognosis	9	–	8	9	7	4	5	7	9	–
"tüdőrák túlélési esélyei"	Disease prognosis	–	–	–	6	3	–	3	–	–	7
"tüdőrák tünetei hátfájás"	Symptoms	7	4	–	–	2	–	–	–	–	–
"tüdőrák végstádium"	Disease prognosis	6	5	9	6	5	5	8	7	6	–
"tüdőgyulladás tünetei"	Other cancer-related information	5	4	8	–	–	5	–	5	–	4
"tüdőrák első jelei"	Symptoms	5	–	3	–	–	–	3	–	–	–
"tüdőrák lelki okai"	Other cancer-related information	2	2	3	–	6	3	6	4	5	6
"tüdőrák utolsó stádiuma"	Disease prognosis	–	–	3	4	–	–	–	1	–	–
"tüdőrák tünetei gyakori kérdések"	Symptoms	–	–	–	–	3	–	–	–	4	–
"tüdőrák 4 stádium"	Disease prognosis	–	–	–	–	1	–	–	–	–	–
"tüdőrák tünetei nőknél"	Symptoms	–	–	–	–	–	1	3	–	5	4
Breast cancer											
"mellrák tünetei"	Symptoms	100	100	100	100	100	100	100	100	100	100
"mellrák jelei"	Symptoms	18	17	16	22	16	18	33	24	12	7
"mellrák fórum"	Other cancer-related information	16	11	13	15	3	5	9	0	6	–
"mellrák elleni kampány"	Other cancer-	12	–	–	–	–	–	–	–	–	–

Search query	Intent category	2013	2014	2015	2016	2017	2018	2019	2020	2021	2022
	related information										
"mammográfia"	Screening and early detection	7	–	–	–	–	–	–	–	–	–
"angelina jolie mellrák"	Other cancer-related information	7	–	–	–	–	–	–	–	–	–
"mellrák elleni kampány facebook"	Other cancer-related information	7	–	–	–	–	–	–	–	–	–
"gyulladásos mellrák tünetei"	Symptoms	6	–	–	2	4	3	–	–	–	–
"mellrák képek"	Other cancer-related information	6	–	3	12	–	7	–	–	–	–
"férfi mellrák"	Other cancer-related information	6	13	–	6	11	8	8	6	10	8
"mellrák lelki okai"	Other cancer-related information	6	8	–	6	6	15	11	12	19	14
"mellrák szűrés"	Screening and early detection	6	–	–	–	–	–	–	–	–	–
"mellrák stádiumai"	Disease prognosis	5	10	6	–	–	4	4	–	–	6
"mellrák fajtái"	Other cancer-related information	5	–	–	–	–	6	9	–	–	–
"mellrák tünetek"	Symptoms	4	4	10	–	3	6	11	2	–	–
"mellrákszűrés"	Screening and early detection	2	–	–	–	–	–	–	–	–	6
"gyulladásos mellrák"	Other cancer-related information	–	8	7	10	19	15	12	8	11	13
"emlőrák"	Other cancer-related information	–	–	12	7	–	–	–	–	–	–
"mellrák tünetei képekkel"	Symptoms	–	–	–	6	9	–	2	5	12	6
"tripla negatív mellrák"	Other cancer-related information	–	–	–	–	–	3	–	4	–	–

Search query	Intent category	2013	2014	2015	2016	2017	2018	2019	2020	2021	2022
"szépségkirálynő mellrák"	Other cancer-related information	–	–	–	–	–	3	–	–	–	–
"mellrák adókedvezmény"	Other cancer-related information	–	–	–	–	–	11	–	–	–	–
"mellrák világnapja"	Other cancer-related information	–	–	–	–	–	–	–	10	5	10
"mellrák világnapja (évszám)"	Other cancer-related information	–	–	–	–	–	–	–	7	–	3
"mellrák elleni világnap"	Other cancer-related information	–	–	–	–	–	–	–	1	–	2
Colorectal cancer											
"vastagbélrák tünetei"	Symptoms	100	100	100	100	100	100	100	100	100	100
"vastagbél"	Other cancer-related information	20	21	24	20	15	26	–	30	32	21
"vastagbélrák szűrés"	Screening and early detection	18	28	17	22	17	26	34	28	20	23
"vastagbélgyulladás"	Other cancer-related information	13	12	2	6	8	2	–	4	3	–
"vastagbélrák tünete"	Symptoms	7	9	10	6	4	7	–	4	–	5
"vastagbélrák tünetek"	Symptoms	7	8	8	5	4	6	4	5	12	3
"végbélrák"	Other cancer-related information	5	9	–	–	8	9	9	6	–	–
"vastagbélrák lelki okai"	Other cancer-related information	3	–	–	9	4	15	20	5	5	5
"vastagbélrák túlélési esélyei"	Disease prognosis	–	–	–	8	6	3	8	9	12	3
"vastagbélrák tünetei nőknél"	Symptoms	–	–	–	–	–	7	–	–	–	–
Prostate cancer											
"prosztatarák tünetei"	Symptoms	100	100	100	100	100	100	100	100	100	100
"prosztata"	Other cancer-related information	80	63	78	92	74	74	41	31	29	43

Search query	Intent category	2013	2014	2015	2016	2017	2018	2019	2020	2021	2022
"prosztatagyulladás"	Other cancer-related information	33	26	21	26	–	–	–	–	–	6
"prosztatrák gyógyítása"	Treatment	10	–	14	–	15	–	–	–	6	12
"prosztatarák szűrés"	Screening and early detection	6	12	21	29	10	30	–	–	–	–
"prosztatarák tünete"	Symptoms	–	–	11	12	–	–	–	–	–	–
"hererák"	Other cancer-related information	–	–	–	17	–	–	–	–	–	–
"prosztatagyulladás tünetei"	Other cancer-related information	–	–	–	–	8	–	–	–	–	7
"prosztatarák stádiumai"	Disease prognosis	–	–	–	–	–	11	5	–	–	3
"prosztata tünetei"	Other cancer-related information	–	–	–	–	–	–	–	24	–	–
Pancreatic cancer											
"hasnyálmirigy"	Other cancer-related information	100	–	100	55	50	–	69	81	–	78
"hasnyálmirigyrák tünetei"	Symptoms	86	100	96	100	100	100	100	–	100	100
"hasnyálmirigy gyulladás"	Other cancer-related information	40	29	65	17	19	31	24	7	–	28
"hasnyálmirigy gyulladás tünetei"	Other cancer-related information	25	16	25	11	9	19	–	86	–	13
"hasnyálmirigyrák tünetek"	Symptoms	12	–	39	–	15	–	–	7	–	–
"hasnyálmirigyrák lelki okai"	Other cancer-related information	7	10	12	–	7	–	–	42	–	14
"hasnyálmirigyrák lefolyása"	Disease prognosis	–	7	19	12	–	9	–	12	–	15
"hasnyálmirigyrák tünet"	Symptoms	–	–	3	4	–	–	–	–	–	–
"hasnyálmirigyrák okai"	Other cancer-related information	–	–	–	21	15	–	7	62	–	–

Search query	Intent category	2013	2014	2015	2016	2017	2018	2019	2020	2021	2022
"esterházy péter"	Other cancer-related information	–	–	–	3	–	–	–	–	–	–
"hasnyálmirigy rák"	Other cancer-related information	–	–	–	–	–	11	24	–	45	13
"hasnyálmirigyrák benedek"	Other cancer-related information	–	–	–	–	–	–	–	100	–	–
"benedek tibor"	Other cancer-related information	–	–	–	–	–	–	–	93	–	–
"benedek tibor hasnyálmirigyrák"	Other cancer-related information	–	–	–	–	–	–	–	88	–	–
"hasnyálmirigy rák benedek tibor"	Other cancer-related information	–	–	–	–	–	–	–	26	–	–
"benedek tibor betegsége"	Other cancer-related information	–	–	–	–	–	–	–	12	–	–
"benedek tibor beteg"	Other cancer-related information	–	–	–	–	–	–	–	8	–	–

RSV: relative search volume. Annual RSV values were obtained from Google Trends™ (GT) "top" related queries for each cancer-related search term. Search queries were classified according to their primary information-seeking intent as symptoms, screening and early detection, treatment, disease prognosis, or other cancer-related information. A dash (–) indicates that the respective query was not identified among the retained top related queries in that year. Hungarian search queries are presented verbatim in quotation marks.
